# Halogenation at the Phenylalanine Residue of Monomethyl Auristatin F Leads to a Favorable *cis*/*trans* Equilibrium and Retained Cytotoxicity

**DOI:** 10.1021/acs.molpharmaceut.1c00342

**Published:** 2021-07-23

**Authors:** Iris K. Sokka, Surachet Imlimthan, Mirkka Sarparanta, Hannu Maaheimo, Mikael P. Johansson, Filip S. Ekholm

**Affiliations:** † Department of Chemistry, University of Helsinki, P.O. Box 55, A. I. Virtasen aukio 1, FI-00014 Helsinki, Finland; ‡ VTT Technical Research Centre of Finland Ltd, P.O. Box 1000, VTT, FI-02044 Espoo, Finland; § CSC, IT Center for Science Ltd., P.O. Box 405, FI-02101 Espoo, Finland

**Keywords:** antibody−drug conjugates, auristatins, cancer therapeutics, structural characterization, NMR-spectroscopy

## Abstract

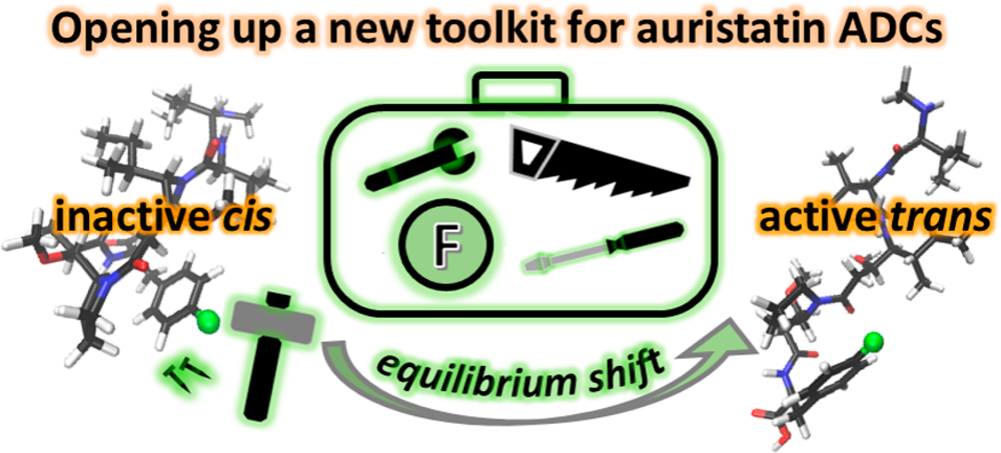

Halogenation can be utilized for the purposes of labeling and molecular imaging, providing a means to, e.g., follow drug distribution in an organism through positron emission tomography (PET) or study the molecular recognition events unfolding by nuclear magnetic resonance (NMR) spectroscopy. For cancer therapeutics, where often highly toxic substances are employed, it is of importance to be able to track the distribution of the drugs and their metabolites in order to ensure minimal side effects. Labeling should ideally have a negligible disruptive effect on the efficacy of a given drug. Using a combination of NMR spectroscopy and cytotoxicity assays, we identify a site susceptible to halogenation in monomethyl auristatin F (MMAF), a widely used cytotoxic agent in the antibody–drug conjugate (ADC) family of cancer drugs, and study the effects of fluorination and chlorination on the physiological solution structure of the auristatins and their cytotoxicity. We find that the cytotoxicity of the parent drug is retained, while the conformational equilibrium is shifted significantly toward the biologically active *trans* isomer, simultaneously decreasing the concentration of the inactive and potentially disruptive *cis* isomer by up to 50%. Our results may serve as a base for the future assembly of a multifunctional toolkit for the assessment of linker technologies and exploring bystander effects from the warhead perspective in auristatin-derived ADCs.

## 1 Introduction

The auristatins are efficient mitosis inhibitors that bind to microtubules and prevent cell proliferation.^1−5^ Since the original discovery of dolastatin 10 in 1987,^6^ the auristatins have attracted considerable interest due to their exceptional cytotoxicity, which is roughly 100–1000 times higher than that of doxorubicin, a previously often employed anticancer therapeutic.^7^ While this potential was intriguing to the scientific community, the high toxicity imposed severe constraints, which limited the practical applicability of auristatins for decades. Still today, the high toxicity is a major factor that needs to be addressed when aiming at auristatin-derived drugs.

The auristatin family of cytotoxic agents has evolved significantly over the last three decades. Notably, they have shaped the modern antibody–drug conjugate (ADC) era.^8^ In fact, the second ADC to be approved for clinical use was brentuximab vedotin in 2011,^9^ featuring the auristatin monomethyl auristatin E (MMAE) as the cytotoxic warhead. Currently, there are multiple different auristatin-containing ADCs approved for clinical use and several more in the development pipeline.^10−12^ The motivation for incorporating auristatins in ADCs is that the powerful cytotoxicity displayed by the auristatins can thereby be harnessed at its full potential by significantly reducing the off-site toxicity due to the highly specific targeting capabilities of monoclonal antibodies.^13^ While this is true in theory, in practice, the auristatin ADCs do display a number of side effects such as neutropenia, neuropathy, thrombocytopenia, and ocular toxicity.^14^ A consequence of the adverse effects may be the reason why these ADCs are, for the most part, not currently considered as the primary cancer treatment options.^2,15−17^

In general, the side effects of ADCs stem from one or more of the three components they are composed of, that is, the antibody, the linker species, or the cytotoxic warhead, either directly or through their metabolic products. Therefore, an essential aspect of the development of improved ADCs is the possibility of monitoring all three components individually in a biological milieu. In this regard, installing a traceable label in the warhead would enable the rapid assessment of linker technologies (factors related to linker stability and release of the warhead) and shed light on the connection between off-site cytotoxicity, the bystander effect, and the location of the cytotoxic agent and its metabolic products. Such a multifunctional tool would provide a sound outlet for assessing safety and functional aspects of ADCs as well as complementing the current assessment models, which evolve around the use of labeled antibodies to a high degree.^18−20^

**Figure 1:**
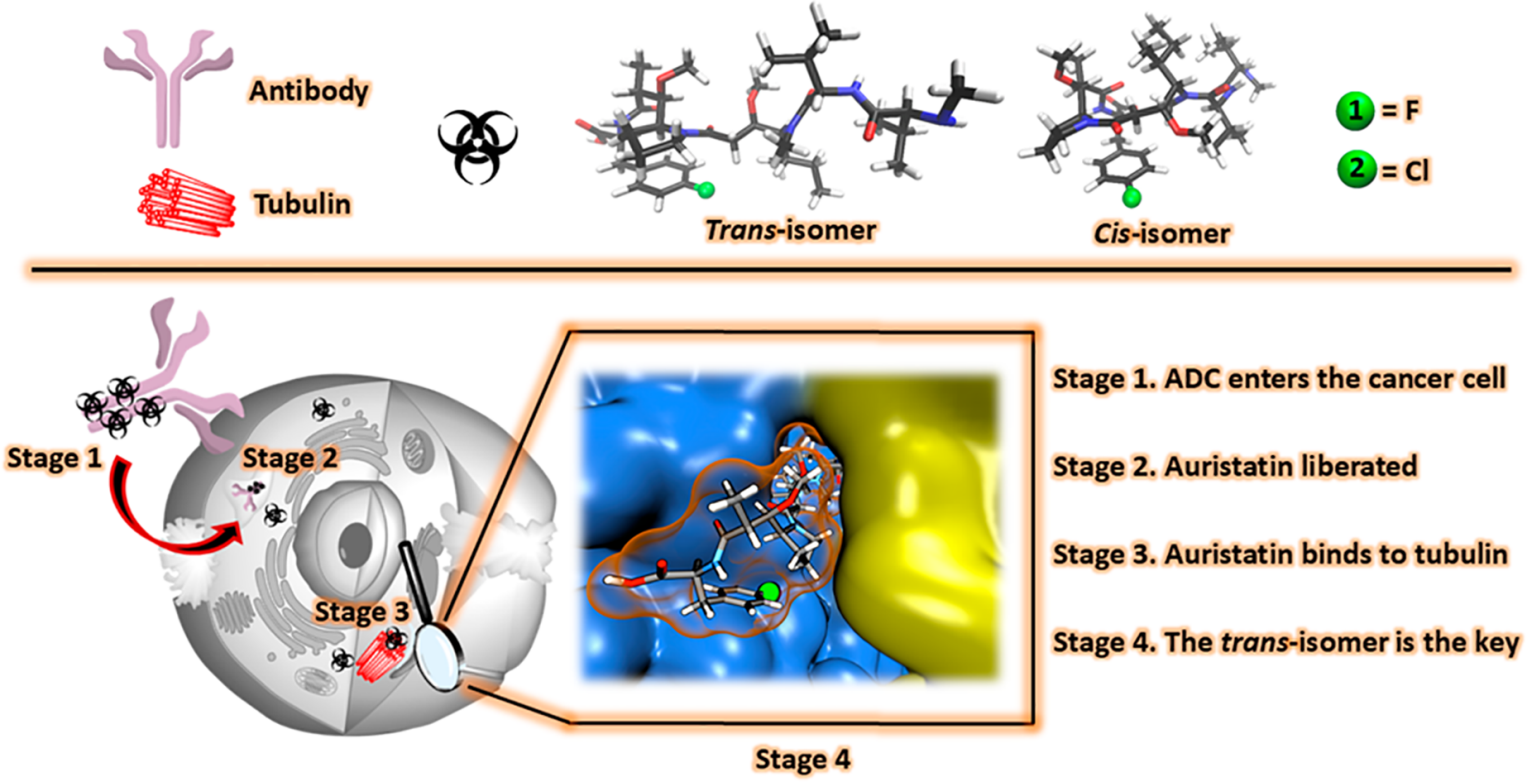
Upper panel: Definitions and the 3D structures of the halogenated auristatins (**1** (F-MMAF); **2** (Cl-MMAF)). Lower panel: The mechanism of ADCs in a biological system is displayed with further emphasis on the biologically active *trans* isomer. The blow-up shows F-MMAF binding to the α and β units of the tubulin dimer (yellow and blue).

When it comes to the auristatins, the construction of a structural analogue containing a label has proved challenging, and the use of ^14^C-^21^ or ^3^H-labeled^22^ auristatins has been the often employed option. This is because structural modifications in the auristatin core are not well tolerated; instead, they are often accompanied by a significant loss of cytotoxicity.^23,24^ During the past few years, we have assessed the solution properties of the auristatins monomethyl auristatin E and F (MMAE, MMAF).^25,26^ The auristatins exist as a mixture of two conformational isomers, denoted *cis* and *trans*. Of these, only the elongated structure of the *trans* isomer fits in the tubulin receptor pocket between the α and β units of the tubulin dimer (see Figure 1) and is considered biologically active. Due to the more compact three-dimensional structure of the *cis* isomer, it is not capable of entering this central binding site. The *cis* isomer can, over the course of hours, isomerize to the *trans* form. This is unfavorable if the drug molecule is no longer internalized in the cancer cells when the activation occurs. In this event, the effects can be lethal to healthy cells within the organism, leading to adverse effects.

To investigate the possibility of shifting the conformational equilibrium, we recently rationally designed auristatin derivatives by halogenation in the norephedrine/phenylalanine residues. High-level quantum chemical modeling suggested that this should lead to improved overall qualities.^27^ In addition to providing a means for inserting a fluorine label in the core structure (or other halogen-based radiolabels), halogenation was shown to potentially shift the *cis*/*trans* equilibrium of the drug significantly toward the biologically active *trans* isomer. This effect was more pronounced in MMAF than in MMAE.^27^

Herein, we continue our investigation by assessing the outcome of halogenation on the *cis*/*trans* equilibrium and solution structure of MMAF and compare the toxicity of the MMAF derivatives to that of the parent molecule. Our results show that halogenation at the *para*-position in the phenylalanine residue of MMAF indeed imposes a desired shift in the *cis*/*trans* equilibrium. Equally importantly, the potency of the parent drug is retained. Altogether, our results open up avenues for the assembly of a functional toolkit that can be utilized in the assessment of linker technologies and gaining insights on the pharmacokinetics and pharmacodynamics properties arising from the warhead in ADCs (e.g., by translational, quantitative, and sensitive PET imaging).

## 2 Experimental Section

### Materials and Chemicals

Murine B16–F10 melanoma (ATCC CRL-6475) and human SKOV3 ovarian adenocarcinoma cell lines (ATCC HTB-77) were obtained from American Type Culture Collection (Manassas, VA, USA). TC-treated cell culturing flasks and 96-well plates were purchased from Corning (Corning, NY, USA). Dulbecco’s modified Eagle’s medium (DMEM), McCoy’s 5a modified medium, Dulbecco’s phosphate buffer saline (10 × DPBS), Hank’s balanced salt solution (1 × HBSS), fetal bovine serum (FBS), GlutaMax (100×), and Penicillin-Streptomycin (10 000 U/ml) were purchased from Gibco (Life Technologies, Carlsbad, CA, USA). The CellTiter-Glo luminescent cell viability assay was acquired from Promega Corporation (Madison, WI, USA). The Pierce BCA Protein Assay Kit was obtained from Thermo Fisher Scientific (Waltham, MA, USA).

### 2.1 NMR Experiments

The NMR samples were prepared by dissolving **1** (F-MMAF) and **2** (Cl-MMAF), respectively, in D_2_O. The NMR experiments were carried out at 37 °C on an 850 MHz Bruker Avance III HD NMR spectrometer equipped with a TCI (H–C/N-D) cryogenic probe. Standard Bruker pulse sequence programs with gradient selection were used. In the 2D TOCSY and the 2D HSQC-TOCSY experiments, dipsi2 spinlocks with durations of 180 and 120 ms, respectively, were used. The ^13^C multiplicity edited HSQC (edHSQC) and the 2D HSQC-TOCSY spectra were acquired using echo/antiecho-TPPI gradient selection, ^1^*J*_CH_ of 145 Hz, and adiabatic decoupling. In the HSQC, the refocusing pulses were also adiabatic. The HMBC experiments were optimized for 8 Hz (62.5 ms) long-range coupling. The mixing time in the ROESY experiments was 800 ms. The NMR spectra were processed in Bruker Topspin 4.0.7.

### 2.2 Cell Viability Studies

The *in vitro* cell cytotoxicity of auristatin compounds (MMAF, **1** (F-MMAF), and **2** (Cl-MMAF)) was studied using a commercial CellTiter-Glo cell viability assay based on luminescent detection of ATP generation in viable cells. B16–F10 and SKOV3 cells were selected as murine and human cancer cell models, respectively. Cells were seeded on a 96-well clear bottom white polystyrene microplate at a density of 5000 cells per well in 100 μL of corresponding media (DMEM for B16–F10 and McCoy’s 5a for SKOV3) supplemented with 1 × GlutaMax, 1% Penicillin-Streptomycin and 10% FBS and allowed to attach overnight. The media was then replaced with 100 μL of MMAF, **1** (F-MMAF), and **2** (Cl-MMAF) in corresponding cell culture media at concentrations of 1 nM, 10 nM, 0.1 μM, 1 μM, and 10 μM, while fresh media and 1% (v/v) Triton X-100 were used as negative and positive controls for cytotoxicity, respectively. The cells were incubated in a temperature- and humidity-controlled incubator (37 °C, 95% relative humidity, and 5% CO_2_) and taken out for analysis at different predetermined time points (24, 48, and 72 h). At each time point, the plate was equilibrated for 30 min to room temperature (RT), then the test solutions were removed, and the cells were washed twice with 100 μL of 1 × DPBS. For the viability assay, 50 μL of 1 × HBSS and CellTiter-Glo cocktail were added to each well. The plates were immediately protected from light with aluminum foil and gently shaken on an orbital shaker for 2 min at RT. The ATP-generated luminescence was measured using a Varioskan LUX multimode microplate reader (Thermo Fisher Scientific, Waltham, MA, USA). The total protein content quantified using the colorimetric bicinchoninic acid (BCA) protein assay in each sample was used to normalize the cell viability results. The BCA assay procedure was carried out according to the manufacturer’s protocol. Briefly, 25 μL of cell lysates from Cell-TiterGlo samples were pipetted to a 96-well clear bottom UV-transparent microplate. Then, 200 μL of working reagent (WR) were added to each well (1:8 ratio). Plates were wrapped with aluminum foil and mixed on an orbital shaker for 30 s before further incubating at 37 °C for 30 min. The absorbance was read at 562 nm, and the protein content was calculated using a bovine serum albumin (BSA) standard curve (0–2000 μg/mL). All experiments were carried out in quadruplicate. The statistical significance of mean cell viability was determined using unpaired Student’s *t*-test compared to the reference compound MMAF and the negative control for cytotoxicity (untreated cells).

## 3 Results and Discussion

**Figure 2:**
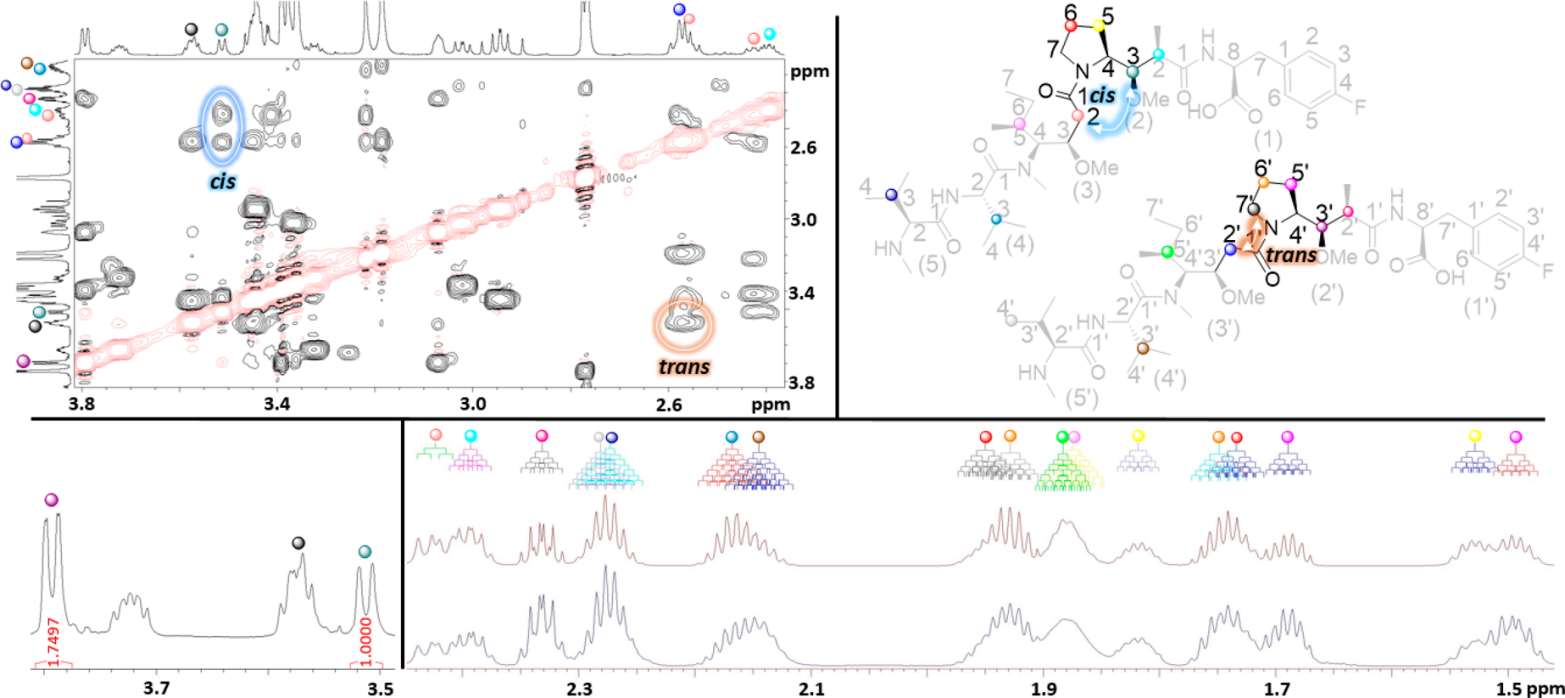
An excerpt of the NMR structural characterization studies. Top left: The 3.80–2.40 region of the ROESY spectra with the ROE-crosspeaks used to identify the *cis*/*trans* isomers highlighted. Top right: The numbering of positions in the isomers of **1** (F-MMAF) are displayed along with the colors used for visualization of key signals in the NMR spectra. The amino acid residues are numbered as (1)/(1′) *p*-Fluorophenylalanine, (2)/(2′) dolaproine, (3)/(3′) dolaisoleuine, (4)/(4′) valine, and (5)/(5′) monomethyl valine for the *cis*/*trans* isomers. Bottom left: Integration values used to determine the *cis*/*trans* ratio in **1**. Bottom right: The 2.45–1.46 ppm region of the ^1^H NMR spectrum highlighting the accuracy of the spectral simulations with the PERCH software (bottom: measured spectrum, top: simulated spectrum).

In order to move toward a functional toolkit featuring radiolabeled auristatins, it is crucial to understand the effects exerted by halogen atoms on the parent molecule as the auristatins have been found to be sensitive to modifications at their structural core. As a result, we here focus on the detailed conformational characterization of the auristatin-derivatives including the assessment of the effects of halogenation on the *cis*/*trans* equilibrium and the cytotoxicity. The *para*-chloro and *para*-fluoro substituted MMAF analogues (see Figure 1) were purchased from Levena Biopharma, and we began by addressing the effects of the halogen atoms on the solution structure and *cis*/*trans* equilibrium. In contrast to the excellent NMR spectroscopic characterization studies performed previously on dolastatin 10,^28,29^ MMAE, and MMAF,^26^ we decided to move one step closer to physiological conditions by performing the structural characterization and conformational analysis in D_2_O at 37 °C. While Benedetti et al. noted that the conformational properties of the auristatins are highly dependent on the solvent,^29^ there is not a single focused study to date, to the best of our knowledge, which has attempted to assess the solution structure of these cytotoxic agents in D_2_O. The reasons are probably related to their marginal aqueous solubility. Nevertheless, the aqueous solubility of the MMAF-derivatives **1** and **2** proved sufficient. During our NMR spectroscopic investigations, we used an 850 MHz NMR instrument and the following set of NMR spectroscopic techniques: 1D ^1^H and ^13^C; 2D COSY (correlation spectroscopy), 2D ^13^C multiplicity edited HSQC (heteronuclear single-quantum coherence, edHSQC), 2D TOCSY (total correlation spectroscopy), 2D HSQC-TOCSY, 2D HMBC (heteronuclear multiple bond correlation), and 2D ROESY (rotating-frame nuclear Overhauser effect spectroscopy). The key methods for identifying and assigning the signals in the complex ^1^H and ^13^C NMR spectra of both *cis* and *trans* isomers of **1** and **2** were high-resolution COSY, TOCSY (2D), HSQC-TOCSY, edHSQC, and HMBC. In addition, the spectral simulation software PERCH (Peak ResearCH) was utilized to analyze coupling constants and patterns.^30^ The PERCH software employs quantum mechanical optimization as part of the iteration process. This aid is required when performing the complete assignation of otherwise challenging NMR spectra containing higher-order effects and severely overlapping signals.^31−33^ A detailed guide to the NMR spectroscopic characterization of auristatins was provided previously,^26^ and the structural characterization flow will therefore not be discussed in detail here. Instead, the focus will be placed on discussing the *cis*/*trans* equilibrium and solution structure of **1** and **2**. The chemical shifts, coupling constants, HMBC, and ROE (rotating-frame nuclear Overhauser effect) correlations which form the basis of the continued discussion are summarized in Supporting Tables 1–4 (see Supporting Information), and an excerpt from the structural characterization part is provided in Figure 2.

In MMAF, the *cis*/*trans* ratio has been determined to be 60:40 in favor of the biologically inactive *cis* isomer. This isomer ratio is not limited to MMAF; other members of the auristatin family display similar ratios regardless of their surroundings as indicated by previous ^1^H NMR measurements in solvents including DMSO, CD_2_Cl_2_, CDCl_3_, and CD_3_OD.^26,28,29^ The energy barrier for conversion of the inactive *cis* isomer to the biologically active *trans* isomer has been calculated to be roughly 101 kJ/mol, which raises concerns on the availability of the active isomer once the drug is released inside the targeted cancer cell. Based on our recent computational predictions,^27^ a halogenation at the *para*-position of phenylalanine in MMAF would exert a significant shift in the *cis*/*trans* ratios and lead to more than 90% of the biologically active *trans* isomer. This might significantly improve the potency of these cytotoxic agents, reduce the required doses, and improve their safety profiles.

With the identification of all signals on the dolaproine and dolaisoleuine residues, the *cis* and *trans* isomers could be identified and their respective ratios determined (Figure 2). In more detail, the identification of the *cis* isomers was based on the ROE correlations between H-3 (2) (dd at 3.51 ppm) and H-2a (3) (d at 2.57 ppm) and the H-4 (2) (ddd at 3.44 ppm) and H-2b (3) (dd at 2.42 ppm) in **1** and H-4 (2) (ddd at 3.21 ppm) and H-2 (3) (d at 2.55 ppm; dd at 2.39 ppm) in **2**. On a related note, the *trans* isomers displayed ROE correlations between H-7′ (2′) (H-7′a: ddd at 3.57 ppm; H-7′b: ddd at 3.45 ppm) and H-2′ (3′) (H-2′a: dd at 2.58 ppm; H-2′b: dd at 2.55 ppm) in **1** and H-7′a (2′) (ddd at 3.60 ppm) and H-2′a (3′) (d at 2.62 ppm) in **2**. These patterns in the dolaproine and dolaisoleuine residues are identical to the ones observed in our previous work on MMAE and MMAF.^26^ Integration of the well-resolved signals in **1** (H-3 (2) (dd at 3.51 ppm) and H-3′ (2′) (dd at 3.79 ppm) and **2** (2-Me (2) (d at 1.24 ppm) and 2′-Me (2′) (d at 1.18 ppm)) gave experimentally determined *cis*/*trans* ratios of 36:64 for **1** and 30:70 for **2** in D_2_O at 37 °C. These values confirm the computationally predicted isomer shift, although the observed shift is somewhat lower than the modeled values. Nevertheless, a significant increase of 60% of the biologically active isomer of **1**, and a 75% increase of the biologically active isomer of **2** is seen; in other words, the concentration of the inactive *cis* isomer is halved.

These experimental results confirm that disruptive elements situated in close proximity to the interior of the contorted *cis* isomer can be used to inflict a considerable shift in the *cis*/*trans* equilibrium in favor of the extended biologically active *trans* isomer.^5^ In order to ascertain that the modifications were not accompanied by other notable structural changes which might impact the cytotoxicity, we performed a detailed investigation of the three-dimensional solution structures of **1** and **2** by ROESY and compared the results to our previous work on MMAF.^26^

On the general whole, the ROE correlations confirmed that the *cis* and *trans* isomers of **1** and **2** are structurally similar to those experimentally determined for MMAF in CD_3_OD^26^ and previously predicted;^27^ i.e., the *cis* isomer forms a contorted structure in which the phenylalanine, dolaproine, dolaisoleuine, and valine residues are spatially adjacent thus forming an interior framework while the *trans* isomer forms an extended structure (Figure 1). The *cis*/*trans* equilibrium is undoubtedly more complex, as exemplified by the signal broadening effects observed in the dolaproine residue of the *cis* isomer (e.g., H-2 (2), H-5 (2), H-6 (2) in both **1** and **2**). These effects were here interpreted as a rapidly interchangeable dynamic state caused by ring puckering. This state would logically be more pronounced in the *cis* isomer due to its contorted nature and increased steric strain. In comparison, the corresponding signals in the *trans* isomer are sharper, which implies that the extended structure does not display similar behavior.

In our previous NMR spectroscopic study on MMAF, we did not observe an ROE correlation between the phenylalanine residue and the dolaproine residue in the *trans* isomer, and open questions regarding the preferred position of the aromatic ring remained. This was despite the computational model available, which predicted that this structural element would be situated beneath the dolaproine residue. In the current work, we observed an ROE correlation between the H-2′ (1′) and H-5′ (2′) and H-8′ (1′) and H-2′ (2′) in both **1** and **2**, thus proving that the computational model was correct. Further evidence was supplied by the change in the chemical shift of the H-4′ (2′) signals which were found to be dependent on the substituent at the *para*-position. This signal appears at 3.66 ppm in MMAF, 3.07 ppm in **1**, and 2.82 ppm in **2**. Since modification at the *para*-position of the phenylalanine residue is not capable of infusing a change in the dolaproine residue through inductive or resonance effects, the only possibility is that it manipulates the electronic surrounding through its spatial arrangement.

As reported recently in our computational study,^27^ we performed F/I-SAPT0 analysis on the intramolecular interactions that relate to the *cis*/*trans* conformational equilibrium. The method allows inspection of the individual factors that affect the interaction energies. Briefly, the attractive intramolecular energy was more pronounced in the *cis* conformers of unsubstituted auristatins. For the halogenated species, the opposite was observed, and especially, the electrostatic interaction was found to be enhanced in the *trans* conformers.

When moving toward a functional auristatin toolkit centered on improved imaging and assessment capabilities, it was important to verify that an eventual radiolabel would not impede the potency of these drugs. On the basis of our conformational assessment of the solution structures of the *cis* and *trans* isomers of **1** and **2** under physiological conditions, they display the characteristic structural features of auristatins and should therefore retain their cytotoxic activity. The conformational shift leading to an increased amount of the *trans* isomer could even have a beneficial effect on the potency. In order to verify our hypothesis, we performed a cytotoxicity assay using two separate cancer cell lines: the murine B16–F10 melanoma cell line and the human SKOV3 ovarian adenocarcinoma cell line. In the cytotoxicity studies, the untreated cell culture medium was used as a negative control for cytotoxicity, 1% Triton X-100 as a positive control, and MMAF as a reference (see Supporting Figure 15). The inclusion of MMAF as a reference was important as these auristatins have a more pronounced hydrophilic character that reduces their cell membrane penetration capabilities and the associated bystander effect.^3,34^ The auristatins were employed in concentrations ranging from 0.001–10 μM, and the cytotoxicity was evaluated at the standard time points of 24, 48, and 72 h.

**Figure 3:**
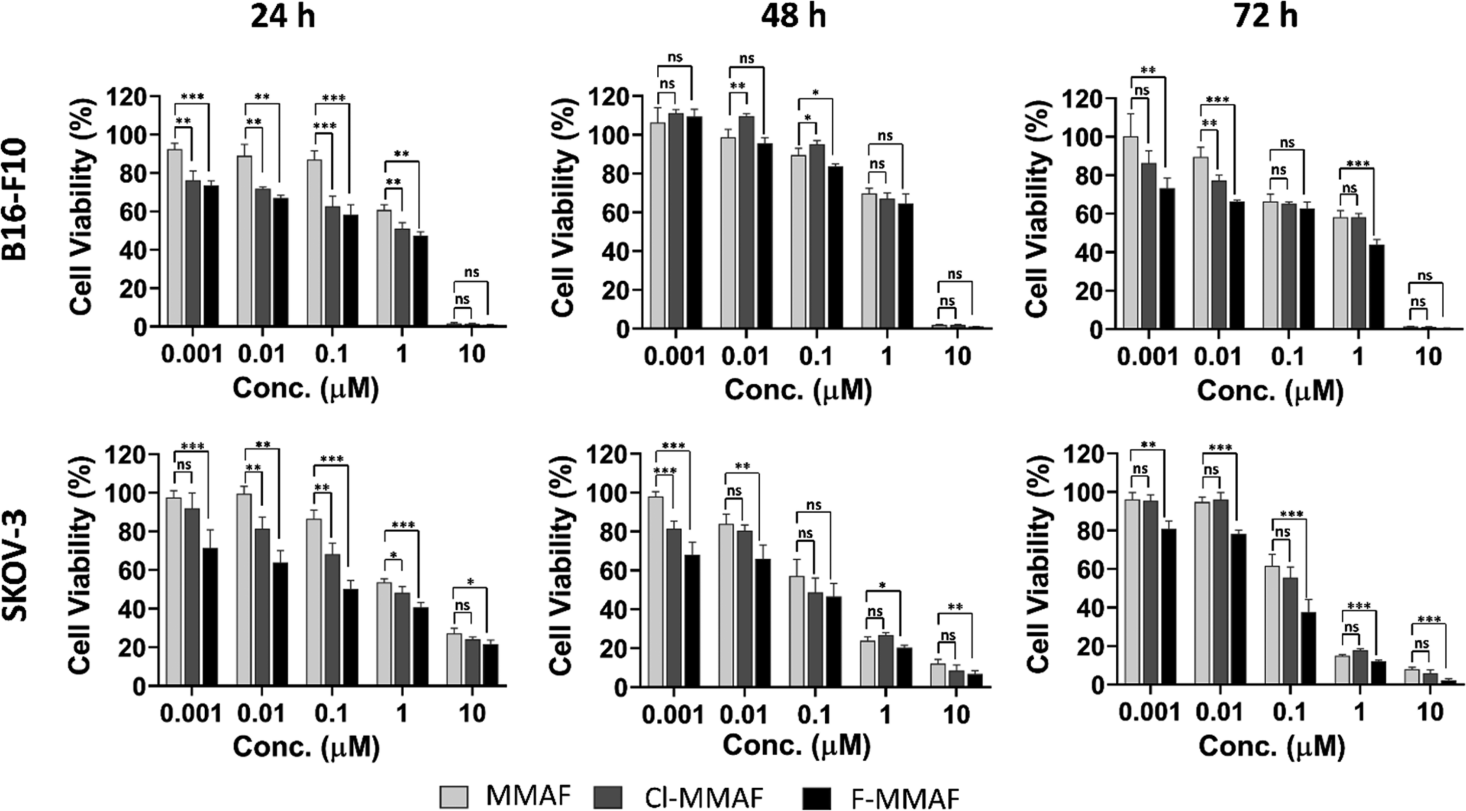
Cell cytotoxicity studies in murine B16–F10 and human SKOV3 cancer cells after incubation with the auristatins; MMAF, **1** (F-MMAF), **2** (Cl-MMAF) at concentrations of 0.001, 0.01, 0.1, 1, and 10 μM for 24, 48, and 72 h. Columns represent the mean ± sd (*n* = 4). The statistical significance of the difference in viability compared to MMAF at the same concentration was determined using unpaired Student’s *t*-test where the significance was set at *p < 0.05, **p < 0.01, and ***p < 0.001.

As seen from the results summarized in Figure 3, MMAF, **1**, and **2** display similar cytotoxicity. This indicates that halogenation at the *para*-position in the phenylalanine residue does not diminish the cytotoxicity of the auristatins. On the contrary, a marginal increase in cytotoxicity is seen for the fluorinated derivative **1** as exemplified by the IC_50_ values, which are 210 nM at 24 h and 80 nM at 72 h for **1** versus 650 nM at 24 h and 110 nM at 72 h for MMAF in the SKOV3 adenocarcinoma cell line and 2.99 μM at 24 h and 1.71 μM at 72 h for **1** versus 2.76 μM at 24 h and 2.04 μM at 72 h for MMAF in the B16–F10 melanoma cell line (see Supporting Figure 16). This being said, the marginal increase in toxicity is not statistically significant, and therefore, the definite conclusion to be drawn is that the potency of all three auristatins is in the same range. When these experimental results are further combined with the computational model, it seems likely that the favorable shift in the *cis*/*trans* equilibrium makes up for the expected decrease in tubulin-binding affinity exerted by the halogen atom. Nevertheless, detailed studies focusing on the connection between the *trans*/*cis* equilibrium, the tubulin-binding affinity, and the potency of auristatins is still required to ascertain these factors. Currently, it is sufficient to note that halogenation at the *para*-position in the phenylalanine residue of the auristatin core is well tolerated.

The well-tolerated halogenation of auristatins showcased herein provides a sound base for a multitude of studies on the behavior of ADCs from the warhead perspective. The modified MMAF-derivative **1** can be used in NMR-based molecular recognition studies in order to provide additional insights on the correlation between the *cis*/*trans* equilibrium, the tubulin-binding affinity, and potency. The radiolabeled counterpart can be utilized to study important ADME properties (absorption, distribution, metabolism, and elimination),^35,36^ assess the stability of linker technologies and even in the clinics for diagnostic purposes.^37^ In addition to using an ^18^F-label, other halogen radiolabels can potentially likewise be employed, e.g., ^123^I, ^124^I, or ^131^I.^35,38,39^ Radiofluorinated and radioiodinated tracers open up possibilities for monitoring the auristatins through both PET and SPECT (single-photon emission computed tomography). This provides a means to assess the behavior of these drugs in a biological setting over a wide range of timeframes (from hours to days/weeks).^35^

## 4 Conclusions

Our interest in the auristatins originates from our initial structural characterization assessment of MMAE and MMAF, which showed that the currently employed warheads exist as an unfavorable mixture of *cis* and *trans* isomers in which the biologically inactive *cis* isomer actually dominates.^26^ While a number of approaches aimed at the development of improved auristatins have been presented over the years, few have resulted in clinical applications. The auristatin core is sensitive toward modifications, and often tinkering with the molecular structure leads to a significant reduction or complete loss of cytotoxicity. Until now, no practical attempt focused on shifting the *cis*/*trans* equilibrium has been reported, with the exception of our recent computational study on the rational design of improved auristatins,^27^ which suggested that there might be significant potential embedded in such an approach.

Here, we showed that halogenation in the phenylalanine residue leads to a more favorable *cis*/*trans* equilibrium with a 60–75% increase in the population of the biologically active *trans* isomer, as predicted by quantum chemical molecular modeling. This corresponds to a reduction in the concentration of the *cis* isomer by up to 50%. Detailed NMR spectroscopic structural characterization revealed that additional effects on the adopted solution conformations were minimal under the modeled physiological conditions. In the cytotoxicity assays, halogenation was found to be well tolerated. The potency of all compounds was in the same range; the fluorinated compound **1** was even found to be marginally more toxic than MMAF. The increase in the population of the active *trans* isomer plausibly counteracts the expected slight decrease in tubulin-binding affinity associated with the halogen atom,^27^ although detailed experimental studies will be required to ascertain these factors.

We emphasize that the halogenated MMAF analogues are more than functional auristatin warheads for the future design of ADCs. They form the very basis of a multifunctional toolkit with a number of important applications in the evaluation of different aspects of auristatin-derived ADCs, such as linker stability, site of linker cleavage, warhead delivery, and metabolic fate. Monitoring these factors from the warhead perspective provides complementary means of addressing safety and potency profiles of ADCs and is a welcomed addition to the current antibody-derived monitoring strategies often employed. The first step on the path to a toolkit aimed at understanding the connections between off-site cytotoxicity, the disputed bystander effect, and the biodistribution of the auristatin warhead and their metabolic products has now been taken.

## Supporting Information Available

NMR spectra, NMR assignment tables, and dose–response diagrams (PDF)

## References

[ref1] Bai R.; Petit G. R.; Hamel E. Dolastatin 10, a powerful cytostatic peptide derived from a marine animal. Inhibition of tubulin polymerization mediated through the vinca alkaloid binding domain. Biochem. Pharmacol. 1990, 39, 1941–1949. 10.1016/0006-2952(90)90613-P. 2353935

[ref2] Senter P. D.; Sievers E. L. The discovery and development of brentuximab vedotin for use in relapsed Hodgkin lymphoma and systemic anaplastic large cell lymphoma. Nat. Biotechnol. 2012, 30, 631–637. 10.1038/nbt.2289. 22781692

[ref3] Doronina S. O.; Mendelsohn B. A.; Bovee T. D.; Cerveny C. G.; Alley S. C.; Meyer D. L.; Oflazoglu E.; Toki B. E.; Sanderson R. J.; Zabinski R. F.; Wahl A. F.; Senter P. D. Enhanced activity of monomethylauristatin F through monoclonal antibody delivery: Effects of linker technology on efficacy and toxicity. Bioconjugate Chem. 2006, 17, 114–124. 10.1021/bc0502917. 16417259

[ref4] Wang Y.; Benz F. W.; Wu Y.; Wang Q.; Chen Y.; Chen X.; Li H.; Zhang Y.; Zhang R.; Yang J. Structural Insights into the pharmacophore of vinca domain inhibitors of microtubules. Mol. Pharmacol. 2016, 89, 233–242. 10.1124/mol.115.100149. 26660762

[ref5] Waight A. B.; Bargsten K.; Doronina S.; Steinmetz M. O.; Sussman D.; Prota A. E. Structural basis of microtubule destabilization by potent auristatin anti-mitotics. PLoS One 2016, 11, e0160890 10.1371/journal.pone.0160890. 27518442 PMC4982639

[ref6] Pettit G. R.; Kamano Y.; Herald C. L.; Tuinman A. A.; Boettner F. E.; Kizu H.; Schmidt J. M.; Baczynskyj L.; Tomer K. B.; Bontems R. J. The Isolation and Structure of a Remarkable Marine Animal Antineoplastic Constituent: Dolastatin 10. J. Am. Chem. Soc. 1987, 109, 6883–6885. 10.1021/ja00256a070.

[ref7] Wu A. M.; Senter P. D. Arming antibodies: Prospects and challenges for immunoconjugates. Nat. Biotechnol. 2005, 23, 1137–1146. 10.1038/nbt1141. 16151407

[ref8] Chari R. V. J.; Miller M. L.; Widdison W. C. Antibody–drug conjugates: An emerging concept in cancer therapy. Angew. Chem., Int. Ed. 2014, 53, 3796–3827. 10.1002/anie.201307628. 24677743

[ref9] Younes A.; Yasothan U.; Kirkpatrick P. Brentuximab vedotin. Nat. Rev. Drug Discovery 2012, 11, 19–20. 10.1038/nrd3629. 22212672

[ref10] Research and Markets Report; Spotlight on Antibody–Drug Conjugates; 4846064, 2019.

[ref11] Chau C. H.; Steeg P. S.; Figg W. D. Antibody–drug conjugates for cancer. Lancet 2019, 394, 793–804. 10.1016/S0140-6736(19)31774-X. 31478503

[ref12] Kaplon H.; Reichert J. M. Antibodies to watch in 2019. mAbs 2019, 11, 219–238. 10.1080/19420862.2018.1556465. 30516432 PMC6380461

[ref13] Schrama D.; Reisfeld R. A.; Becker J. C. Antibody targeted drugs as cancer therapeutics. Nat. Rev. Drug Discovery 2006, 5, 147–159. 10.1038/nrd1957. 16424916

[ref14] Donaghy H. Effects of antibody, drug and linker on the preclinical and clinical toxicities of antibody–drug conjugates. mAbs 2016, 8, 659–671. 10.1080/19420862.2016.1156829. 27045800 PMC4966843

[ref15] Markham A. Belantamab Mafodotin: First Approval. Drugs 2020, 80, 1607–1613. 10.1007/s40265-020-01404-x. 32936437

[ref16] Hanna K. S. Clinical Overview of Enfortumab Vedotin in the Management of Locally Advanced or Metastatic Urothelial Carcinoma. Drugs 2020, 80, 1–7. 10.1007/s40265-019-01241-7. 31823332

[ref17] Deeks E. D. Polatuzumab Vedotin: First Global Approval. Drugs 2019, 79, 1467–1475. 10.1007/s40265-019-01175-0. 31352604 PMC6794237

[ref18] Adumeau P.; Vivier D.; Sharma S. K.; Wang J.; Zhang T.; Chen A.; Agnew B. J.; Zeglis B. M. Site-Specifically Labeled Antibody–Drug Conjugate for Simultaneous Therapy and ImmunoPET. Mol. Pharmaceutics 2018, 15, 892–898. 10.1021/acs.molpharmaceut.7b00802. PMC6466629 29356543

[ref19] Xu H.; Gan L.; Han Y.; Da Y.; Xiong J.; Hong S.; Zhao Q.; Song N.; Cai X.; Jiang X. Site-specific labeling of an anti-MUC1 antibody: probing the effects of conjugation and linker chemistry on the internalization process. RSC Adv. 2019, 9, 1909–1917. 10.1039/C8RA09902B. PMC9059757 35516120

[ref20] Riedl T.; van Boxtel E.; Bosch M.; Parren P. W. H. I.; Gerritsen A. F. High-Throughput Screening for Internalizing Antibodies by Homogeneous Fluorescence Imaging of a pH-Activated Probe. J. Biomol. Screening 2016, 21, 12–23. 10.1177/1087057115613270. PMC4708616 26518032

[ref21] Okeley N. M.; Miyamoto J. B.; Zhang X.; Sanderson R. J.; Benjamin D. R.; Sievers E. L.; Senter P. D.; Alley S. C. Alley Intracellular Activation of SGN-35, a Potent Anti-CD30 Antibody-Drug Conjugate. Clin. Cancer Res. 2010, 16, 888–897. 10.1158/1078-0432.CCR-09-2069. 20086002

[ref22] Kim K. M.; McDonagh C. F.; Westendorf L.; Brown L. L.; Sussman D.; Feist T.; Lyon R.; Alley S. C.; Okeley N. M.; Zhang X.; Thompson M. C.; Stone I.; Gerber H.-.; Carter P. J. Anti-CD30 diabody-drug conjugates with potent antitumor activity. Mol. Cancer Ther. 2008, 7, 2486–2497. 10.1158/1535-7163.MCT-08-0388. 18723494

[ref23] Miyazaki K.; Kobayashi M.; Natsume T.; Gondo M.; Mikami T.; Sakakibara K.; Tsukagoshi S. Synthesis and Antitumor Activity of Novel Dolastatin 10 Analogs. Chem. Pharm. Bull. 1995, 43, 1706–1718. 10.1248/cpb.43.1706. 8536345

[ref24] Maderna A.; Leverett C. A. Recent advances in the development of new auristatins: Structural modifications and application in antibody drug conjugates. Mol. Pharmaceutics 2015, 12, 1798–1812. 10.1021/mp500762u. 25697404

[ref25] Ekholm F.; Ruokonen S.; Redón M.; Pitkänen V.; Vilkman A.; Saarinen J.; Helin J.; Satomaa T.; Wiedmer S. Hydrophilic Monomethyl Auristatin E Derivatives as Novel Candidates for the Design of Antibody-Drug Conjugates. Separations 2019, 6, 1 10.3390/separations6010001.

[ref26] Johansson M. P.; Maaheimo H.; Ekholm F. S. New insight on the structural features of the cytotoxic auristatins MMAE and MMAF revealed by combined NMR spectroscopy and quantum chemical modelling. Sci. Rep. 2017, 7, 15920 10.1038/s41598-017-15674-1. 29162861 PMC5698355

[ref27] Sokka I. K.; Ekholm F. S.; Johansson M. P. Increasing the Potential of the Auristatin Cancer-Drug Family by Shifting the Conformational Equilibrium. Mol. Pharmaceutics 2019, 16, 3600–3608. 10.1021/acs.molpharmaceut.9b00437. PMC6750905 31199662

[ref28] Alattia T.; Roux F.; Poncet J.; Cavé A.; Jouin P. Conformational study of dolastatin 10. Tetrahedron 1995, 51, 2593–2604. 10.1016/0040-4020(95)00008-V.

[ref29] Benedetti E.; Carlomagno T.; Fraternali F.; Hamada Y.; Hayashi K.; Paolillo L.; Shioiri T. Conformational analysis of dolastatin 10: An nmr and theoretical approach. Biopolymers 1995, 36, 525–538. 10.1002/bip.360360414. 7578946

[ref30] Laatikainen R.; Niemitz M.; Weber U.; Sundelin J.; Hassinen T.; Vepsäläinen J. General Strategies for Total-Lineshape-Type Spectral Analysis of NMR Spectra Using Integral-Transform Iterator. J. Magn. Reson., Ser. A 1996, 120, 1–10. 10.1006/jmra.1996.0094.

[ref31] Ekholm F. S.; Sinkkonen J.; Leino R. Fully deprotected β-(1→2)-mannotetraose forms a contorted α-helix in solution: convergent synthesis and conformational characterization by NMR and DFT. New J. Chem. 2010, 34, 667–675. 10.1039/b9nj00702d.

[ref32] Matović J.; Järvinen J.; Sokka I. K.; Imlimthan S.; Raitanen J.; Montaser A.; Maaheimo H.; Huttunen K. M.; Peräniemi S.; Airaksinen A. J.; Sarparanta M.; Johansson M. P.; Rautio J.; Ekholm F. S. Exploring the Biochemical Foundations of a Successful GLUT1-Targeting Strategy to BNCT: Chemical Synthesis and In Vitro Evaluation of the Entire Positional Isomer Library of ortho-Carboranylmethyl-Bearing Glucoconjugates. Mol. Pharmaceutics 2021, 18, 285–304. 10.1021/acs.molpharmaceut.0c00917. 33390018

[ref33] Viitaja T.; Raitanen J.; Moilanen J.; Paananen R. O.; Ekholm F. S. The Properties and Role of O-Acyl-ω-hydroxy Fatty Acids and Type I-St and Type II Diesters in the Tear Film Lipid Layer Revealed by a Combined Chemistry and Biophysics Approach. J. Org. Chem. 2021, 86, 4965–4976. 10.1021/acs.joc.0c02882. 33729799 PMC8041317

[ref34] Kung Sutherland M. S.; Sanderson R. J.; Gordon K. A.; Andreyka J.; Cerveny C. G.; Yu C.; Lewis T. S.; Meyer D. L.; Zabinski R. F.; Doronina S. O.; Senter P. D.; Law C.-.; Wahl A. F. Lysosomal trafficking and cysteine protease metabolism confer target-specific cytotoxicity by peptide-linked anti-CD30-auristatin conjugates. J. Biol. Chem. 2006, 281, 10540–10547. 10.1074/jbc.M510026200. 16484228

[ref35] Kumar K.; Ghosh A. Radiochemistry, Production Processes, Labeling Methods, and ImmunoPET Imaging Pharmaceuticals of Iodine-124. Molecules 2021, 26, 414 10.3390/molecules26020414. PMC7830191 33466827

[ref36] Lockley W. J. S.; McEwen A.; Cooke R. Tritium: a coming of age for drug discovery and development ADME studies. J. Labelled Compd. Radiopharm. 2012, 55, 235–257. 10.1002/jlcr.2928.

[ref37] Gallamini A.; Zwarthoed C.; Borra A. Positron emission tomography (PET) in oncology. Cancers 2014, 6, 1821–1889. 10.3390/cancers6041821. 25268160 PMC4276948

[ref38] Hicks R. J.; Hofman M. S. Is there still a role for SPECT–CT in oncology in the PET–CT era?. Nat. Rev. Clin. Oncol. 2012, 9, 712–720. 10.1038/nrclinonc.2012.188. 23149896

[ref39] Conti M.; Eriksson L. Physics of pure and non-pure positron emitters for PET: a review and a discussion. EJNMMI Physics 2016, 3, 8 10.1186/s40658-016-0144-5. 27271304 PMC4894854

