# Supplementary material for: Halogenation at the Phenylalanine Residue of Monomethyl Auristatin F Leads to a Favorable cis/trans Equilibrium and Retained Cytotoxicity
Source: Mol Pharm. 2021 Jul 23;18(8):3125–31. doi: 10.1021/acs.molpharmaceut.1c00342 (PMC8397390; doi:10.1021/acs.molpharmaceut.1c00342)
Supplement: Supplementary file 1 — mp1c00342_si_001.pdf [file mp1c00342_si_001.pdf]

SUPPORTING INFORMATION

Halogenation at the Phenylalanine Residue of Monomethyl Auristatin F Leads to a Favorable *cis/trans* Equilibrium and Retained Cytotoxicity

Iris K. Sokka,[1] Surachet Imlimthan,[1] Mirkka Sarparanta,[1] Hannu Maaheimo,[2] Mikael P. Johansson[1,3,*] and Filip S. Ekholm[1,*]

[1] Department of Chemistry, University of Helsinki, PO Box 55, A. I. Virtasen aukio 1, FI-00014 Helsinki, Finland.

[2] VTT Technical Research Centre of Finland Ltd, PO Box 1000, FI-02044 VTT, Espoo, Finland.

[3] CSC – IT Center for Science Ltd., P.O. Box 405, FI-02101 Espoo, Finland.

Contact: mikael.johansson@csc.fi; filip.ekholm@helsinki.fi

Table of contents:

1. NMR characterization data………………………………………………page 3

2. Selected NMR spectra ………………………………………………………..page 11

3. Supporting material related to cytotoxicity studies…………….page 18

1. NMR characterization data

Supporting Table 1. Summary of the NMR results of compound **1A** (*cis*-conformer of F-MMAF) measured at 37°C in D_2O with a Bruker 850 MHz instrument. Chemical shifts are expressed in ppm using solvent residual peaks as an internal reference. Coupling patterns are given as d (doublet), t (triplet), q (quartet), m (multiplet) etc. and the approximate coupling constants are provided only once when first encountered.

POSITION	δ ^{13}C	δ ^{1}H (*J*, HZ)	HMBC (H → C)-CORRELATIONS[A]	ROESY CORRELATIONS[A]
**1 (1)**	**133**	**–**	**–**	**–**
2 (1)	130.6	~ 7.35 (m)	arom. C	7(1), 8 (1), 5 (2), (3)/(4)-NCH_3, 3-CH_3(4)
3 (1)	~ 115	~7.20 – 7.00 (m)	arom. C	n.d.
4 (1)	~ 161	–	–	–
5 (1)	~ 115	7.20 – 7.00 (m)	arom. C	7(1), 8 (1), 5 (2), (3)/(4)-NCH_3, 3-CH_3(4)
6 (1)	130.6	~ 7.35 (m)	arom. C	7(1), 8 (1), 5 (2), (3)/(4)-NCH_3, 3-CH_3(4)
7 (1)	35.7	3.37 (H-7a, dd, $J_{7a,8}$ = 4.0, $J_{7a,7b}$ = –14.6 Hz)<br>3.02 (H-7b, dd, $J_{7b,8}$ = 10.6 Hz)	arom C., 8-CO_2H (1), 8 (1)	7a (1) ↔ 7b (1)
8 (1)	53.8	4.71 (dd)	7 (1), 8-CO_2H (1), 1 (2)	7a (1), aliphatic
8-CO_2H (1)	175.3	–	–	–
**1 (2)**	**176.1**	**–**	**–**	**–**
2 (2)	43.8	2.40 (dq, $J_{2,2\text{-}CH3}$ = 6.8, $J_{2,3}$ = 9.7 Hz)	1 (2), 3 (2), 2-CH_3 (2)	5a (2), 2-CH_3 (2)
2-CH_3 (2)	14.3	1.26 (d)	1 (2), 2 (2), 3 (2)	2 (2), 3 (2), 3-OCH_3 (2)
3 (2)	85.8	3.51 (dd, $J_{3,4}$ = 1.0 Hz)	2 (2), 2-CH_3 (2), 4 (2), 5 (2)	2 (2), 2-CH_3 (2), 5a (2), 2a (3), 3-OCH_3 (2)
3-OCH_3 (2)	61.6	3.44 (s)	3 (2)	n.d.
4 (2)	59.7	3.44 (ddd, $J_{4,5a}$ = 6.5, $J_{4,5b}$ = 7.6 Hz)	3 (2), 5 (2)	arom. H, 2b (3), 5 (2)
5 (2)	25.3	1.82 (H-5a, dddd, $J_{5a,6a}$ = 4.6, $J_{5a,6b}$ = 6.5, $J_{5a,5b}$ = –12.4 Hz)<br>1.52 (H-5b, dddd, $J_{5b,6a}$ = 6.3, $J_{5b,6b}$ = 8.0 Hz)	3 (2), 6 (2), 7 (2)	5a (2) ↔ 5b (2)<br>5a (2): 7b (2)
6 (2)	23.0	1.95 (H-6a, dddd, $J_{6a,7a}$ = 7.8, $J_{6a,7b}$ = 8.2, $J_{6a,6b}$ = –12.0 Hz)<br>1.73 (H-6b, dddd, $J_{6b,7a}$ = 5.8, $J_{6b,7b}$ = 7.5 Hz)	n.d.	n.d.

7 (2)	47.4	3.72 (H-7a, ddd, $J_{7a,7b}$ = –11.5 Hz)<br>3.33 (H-7b, ddd)	4 (2), 5 (2), 6 (2), 1 (3)	7a (2): 6b (2)<br>7b (2): 5a (2), 5b (2), 6a (2)
**1 (3)**	**171.7**	**–**	**–**	**–**
2 (3)	35.6	2.57 (H-2a, d, $J_{2a,2b}$ = –15.7 Hz)<br>2.42 (H-2b, dd, $J_{2b,3}$ = 10.0 Hz)	1 (3), 3 (3)	2a (3) ↔ 2b (3)<br>2b (3): 3 (3), (3)/(4)-N-CH_3
3 (3)	78.0	4.18 (ddd, $J_{3,5}$ = –1.6, $J_{3,4}$ = 5.4 Hz)	1 (3), 2 (3), 3-OCH_3 (3)	2 (3), 3-OCH_3 (3), 5 (3), (3)/(4)-N-CH_3, aliphatic
3-OCH_3 (3)	57.9	3.38 (s)	3 (3)	n.d.
4 (3)	57.6	4.75 (dd, $J_{4,5}$ = 10.2 Hz)	3 (3), 5 (3)	2 (3), 5 (3), 6a (3), 7 (3)
5 (3)	~ 32.4	1.88 (ddddq, $J_{5,6a}$ = 6.5, $J_{5,6b}$ = 6.5, $J_{5,5\text{-}CH3}$ = 6.5 Hz)	n.d.	3 (3), 6a (3), 7 (3), (3)/(4)-N-CH_3
5-CH_3 (3)	15.0	1.02 (d)	n.d.	n.d.
6 (3)	25.7	1.40 (H-6a, ddq, $J_{6a,7}$ = 7.3, $J_{6a,6b}$ = –12.0 Hz)<br>~ 1.03 (H-6b, ddq, $J_{6b,7}$ = 7.5 Hz)	4 (3), 5 (3), 7 (3)	6a (3): 3 (3), 4 (3), 5 (3), 7 (3), (3)/(4)-N-CH_3, aliphatic
7 (3)	9.9	0.89 (dd)	5 (3), 6 (3)	4 (3), 5 (3), 6 (3), (3)/(4)-N-CH_3
**(3)/(4)-N-CH_3**	**~ 32.3**	**3.22 (s)**	1 (4), 2 (4)	arom. H, 3-OCH_3 (2), 3 (2), 2 (3), 3 (3), 5 (3), 6 (3), 7 (3), 2 (4), 3 (4)
**1 (4)**	**174.0**	**–**	**–**	**–**
2 (4)	55.8	4.84 (d, $J_{2,3}$ = 8.5 Hz)	1 (4), 3 (4), 4 (4), 3-CH_3 (4), 1 (5)	(3)/(4)-N-CH_3, 3 (4), aliphatic, (5)-N-CH_3
3 (4)	~ 30.0	2.17 (dqq, $J_{3,3\text{-}CH3}$ = 6.8, $J_{3,4}$ = 6.9 Hz)	1 (4), 2 (4), 4 (4), 3-CH_3 (4)	(3)/(4)-N-CH_3, 2 (4), aliphatic,
3-CH_3 (4)	18.4	1.12 (d)	2 (4), 3 (4), 4 (4)	n.d.
4 (4)	18.0 – 17.0	1.06 (d)	n.d.	n.d.
**1 (5)**	**167.2**	**–**	**–**	**–**
2 (5)	67.0	3.84 (d, $J_{2,3}$ = 5.8 Hz)	1 (5), 3 (5), 4 (5), 3-CH_3 (5), (5)-N-CH_3	2 (4), 3 (5), aliphatic, (5)-N-CH_3
3 (5)	~ 30.0	~ 2.27 (dqq, $J_{3,3\text{-}CH3}$ = 6.9, $J_{3,4}$ = 7.0 Hz)	1 (5), 2 (5), 4 (5), 3-CH_3 (5)	2 (5), (5)-N-CH_3
3-CH_3 (5)	18.0 – 17.0	1.10 (d)	2 (5), 3 (5), 4 (5)	n.d.
4 (5)	18.0 – 17.0	1.03 (d)	n.d.	n.d.
**(5)-N-CH_3**	**32.2**	**2.77 (s)**	2 (5)	2 (4), 2 (5), 3 (5), aliphatic

[A] The HMBC and ROE correlations were not determined in crowded areas of the spectrum where the uncertainty was high, if the ROE-correlations were in the aliphatic region it is mentioned in the table.

Supporting Table 2. Summary of the NMR results of compound **1B** (*trans*-conformer of F-MMAF) measured at 37°C in D_2O with a Bruker 850 MHz instrument. Chemical shifts are expressed in ppm using solvent residual peaks as an internal reference. Coupling patterns are given as d (doublet), t (triplet), q (quartet), m (multiplet) etc. and the approximate coupling constants are provided only once when first encountered.

POSITION	δ ^{13}C	δ ^{1}H (*J*, HZ)	HMBC (H → C)-CORRELATIONS[A]	ROESY CORRELATIONS[A]
**1′ (1′)**	**~ 133.0**	**–**	**–**	**–**
2′ (1′)	130.6	~ 7.35 (m)	arom. C	7′ (1′), 8′ (1′), 5′ (2′)
3′ (1′)	~ 115.0	7.20 – 7.00 (m)	arom. C	7′ (1′), 4′ (2′), 5′ (2′), 6′ (2′), 3′-OCH_3 (2′)
4′ (1′)	~ 161.0	–	–	–
5′ (1′)	~ 115.0	7.20 – 7.00 (m)	arom. C	7′ (1′), 4′ (2′), 5′ (2′), 6′ (2′), 3′-OCH_3 (2′)
6′ (1′)	130.6	~ 7.35 (m)	arom. C	7′ (1′), 8′ (1′), 5′ (2′)
7′ (1′)	35.8	3.44 (H-7′a, dd, $J_{7'a,8'}$ = 4.7, $J_{7'a,7'b}$ = –14.0 Hz)<br>2.94 (H-7′b, dd, $J_{7'b,8'}$ = 11.7 Hz)	1′ (1′), 8′-CO_2H (1′), 8′ (1′)	7′a ↔ 7′b
8′ (1′)	53.3	4.92 (dd)	7′ (1′), 8′-CO_2H (1′), 1′ (2′)	arom. H, 7′ (1′), 2′-CH_3 (2′), 4′ (2′)
8′-CO_2H (1′)	175.3	**–**	**–**	–
**1′ (2′)**	**176.3**	**–**	**–**	–
2′ (2′)	44.1	2.33 (dq, $J_{2'a,2'\text{-}CH3}$ = 6.8, $J_{2',3'}$ = 9.7 Hz)	1′ (2′), 3′ (2′), 4′ (2′), 2′-CH_3 (2′)	3′ (2′), 3′-OCH_3 (2′), 4′ (2′), 5′ (2′)
2′-CH_3 (2′)	13.7	1.19 (d)	1′ (2′), 2′ (2′), 3′ (2′)	2′ (2′), 3′ (2′), 3′-OCH_3 (2′), 4′ (2′)
3′ (2′)	82.6	3.79 (dd, $J_{3',4'}$ = 1.8 Hz)	1′ (2′), 2′ (2′), 2′-CH_3 (2′), 3′-OCH_3 (2′), 4′ (2′), 5′ (2′)	2′ (2′), 2′-CH_3 (2′), 3′-OCH_3 (2′), 4′ (2′), 5′a (2′), 6′a (2′)
3′-OCH_3 (2′)	61.1	3.39 (s)	3′ (2′)	n.d.
4′ (2′)	59.2	3.07 (ddd, $J_{4',5'b}$ = 5.9, $J_{4',5'a}$ = 6.3 Hz)	3′ (2′), 5′ (2′), 7′ (2′)	arom. H, 8′ (1′), 2′ (2′),2′-CH_3 (2′), 3′ (2′), 5′ (2′), 7′a (2′)
5′ (2′)	24.3	1.70 (H-5′a, dddd, $J_{5'a,6'b}$ = 5.6, $J_{5'a,6'a}$ = 7.7, $J_{5'a,5'b}$ = –12.7 Hz)<br>1.50 (H-5′b, dddd, $J_{5'b,6'b}$ = 5.8, $J_{5'b,6'a}$ = 7.0 Hz)	3′ (2′), 4′ (2′), 6′ (2′), 7′ (2′)	5′a ↔ 5′b
6′ (2′)	24.2	1.92 (H-6′a, dddd, $J_{6'a,7'b}$ = 5.6, $J_{6'a,7'a}$ = 6.3, $J_{6'a,6'b}$ = –13.7 Hz)<br>1.74 (H-6′b, dddd, $J_{6'b,7'b}$ = 6.8, $J_{6'b,7'a}$ = 7.0 Hz)	n.d.	n.d.
7′ (2′)	48.2	3.57 (H-7′a, ddd, $J_{7'a,7'b}$ = –10.5 Hz)<br>3.45 (H-7′b, ddd)	4′ (2′), 5′ (2′), 6′ (2′), 1′ (3′)	4′ (2′), 5′b (2′), 6′ (2′), 2′ (3′), aliphatic

**1′ (3′)**	**170.9**	–	–	–
2′ (3′)	36.7	2.58 (H-2′a, dd, $J_{2'a,3'}$ = 1.7, $J_{2'a,2'b}$ = –16.0 Hz) 2.55 (H-2′b, dd, $J_{2'b,3'}$ = 8.8 Hz)	1′ (3′), 3′ (3′)	7′a (2′), 3′ (3′), 3′-OCH_3 (3′), 5′ (3′), (3′)/(4′)-N-CH_3, aliphatic
3′ (3′)	78.0	4.16 (dddd, $J_{3',5'}$ = –2.5, $J_{3',4'}$ = 5.0 Hz)	n.d.	2′ (3′), 3′-OCH_3 (3′), 5′ (3′), (3′)/(4′)-N-CH_3, aliphatic
3′-OCH_3 (3′)	57.6	3.36 (s)	3′ (3′)	n.d.
4′ (3′)	~ 57.8	4.70 (dd, $J_{4',5'}$ = 4.7 Hz)	1′ (4′)	n.d.
5′ (3′)	~ 32.4	1.88 (dddq, $J_{5',5'\text{-}CH3}$ = 6.5, $J_{5',6'a}$ = 6.5, $J_{5',6'b}$ = 6.5 Hz)	n.d.	2′ (3′), 3′ (3′), 6′a (3′), 7′ (3′), (3′)/(4′)-N-CH_3
5′-CH_3 (3′)	15.3	~ 1.06 (d)	n.d.	n.d.
6′ (3′)	25.7	1.40 (H-6′a, ddq, $J_{6'a,7'}$ = 7.5, $J_{6',6'b}$ = –12.0 Hz) 1.03 (H-6′a, ddq, $J_{6'b,7'}$ = 7.4 Hz)	4′ (3′), 5′ (3′), 5′-CH_3 (3′), 7′ (3′)	6′a (3′): 3′ (3′), 4′ (3′), 5′ (3′), 7′ (3′), (3′)/(4′)-N-CH_3, aliphatic
7′ (3′)	10.1	0.90 (dd)	5′ (3′), 6′ (3′)	5′ (3′), 6′ (3′)
**(3′)/(4′)-N-CH_3**	**~ 32.3**	**3.19 (s)**	4′ (3′), 1′ (4′)	2′ (3′), 3′ (3′), 6′a (3′), 7′ (3′), 2′ (4′), 3′ (4′)
**1′ (4′)**	**174.1**	–	–	–
2′ (4′)	55.8	4.82 (d, $J_{2',3'}$ = 9.0 Hz)	3′ (4′), 3′-CH_3 (4′), 4′ (4′), 1′ (5′)	(3′)/(4′)-N-CH_3, 3′ (4′), aliphatic, (5′)-N-CH_3, 2′ (5′)
3′ (4′)	~ 30.0	2.14 (dqq, $J_{3',4'}$ = 6.5, $J_{3',3'\text{-}CH3}$ = 7.0 Hz)	2′ (4′), 3′-CH_3 (4′), 4′ (4′)	(3′)/(4′)-N-CH_3, 2′ (4′), aliphatic,
3′-CH_3 (4′)	~ 17.9	~ 1.07 (d)	n.d.	n.d.
4′ (4′)	~ 17.9	~1.05 (d)	n.d.	n.d.
**1′ (5′)**	**167.2**	–	–	–
2′ (5′)	67.0	3.84 (d, $J_{2',3'}$ = 5.5 Hz)	1′ (5′), 3′ (5′), 3′-CH_3 (5′), 4′ (5′), (5′)-N-CH_3	2′ (4′), 3′ (5′), aliphatic, (5′)-N-CH_3
3′ (5′)	30.0	2.27 (dqq, $J_{3',4'}$ = 6.5, $J_{3',3'\text{-}CH3}$ = 6.8 Hz)	n.d.	2′ (5′), (5′)-N-CH_3, aliphatic
3′-CH_3 (5′)	17.9	1.10 (d)	2′ (5′), 3′ (5′), 4′ (5′)	n.d.
4′ (5′)	17.2	1.03 (d)	n.d.	n.d.
**(5′)-N-CH_3**	**32.3**	**2.77 (s)**	2′ (5′)	2′ (4′), 2′ (5′), 3′ (5′), aliphatic

[A] The HMBC and ROE correlations were not determined in crowded areas of the spectrum where the uncertainty was high, if the ROE-correlations were in the aliphatic region it is mentioned in the table.

Suppprting Table 3. Summary of the NMR results of compound **2A** (*cis*-conformer of Cl-MMAF) measured at 37°C in D_2O with a Bruker 850 MHz instrument. Chemical shifts are expressed in ppm using solvent residual peaks as an internal reference. Coupling patterns are given as d (doublet), t (triplet), q (quartet), m (multiplet) etc. and the approximate coupling constants are provided only once when first encountered.

POSITION	δ ^{13}C	δ ^{1}H (*J*, HZ)	HMBC (H → C)-CORRELATIONS[A]	ROESY CORRELATIONS[A]
**1 (1)**	**136.0**	**–**	**–**	**–**
2 (1)	130.5	7.35 – 7.30 (m)	arom. C	7 (1), 8 (1), 4 (2), (3)/(4)-N-CH_3, 3-CH_3(4)
3 (1)	128.8	7.44 – 7.35 (m)	arom. C	5b (2), 2 (3), (3)/(4)-N-CH_3, 3-CH_3(4)
4 (1)	132.4	–	–	–
5 (1)	128.8	7.44 – 7.35 (m)	arom. C	5b (2), 2 (3), (3)/(4)-N-CH_3, 3-CH_3(4)
6 (1)	130.5	7.35 – 7.30 (m)	arom. C	7 (1), 8 (1), 4 (2), (3)/(4)-N-CH_3, 3-CH_3(4)
7 (1)	35.9	3.41 (H-7a, dd, $J_{7a,8}$ = 5.4, $J_{7a,7b}$ = –14.6 Hz)<br>2.98 (H-7b, dd, $J_{7b,8}$ = 11.4 Hz)	arom C., 1 (1), 8 (1), 8-CO_2H (1)	7a (1) ↔ 7b (1)<br>7b (1): 8 (1)
8 (1)	53.2	4.80 (dd)	7 (1), 8-CO_2H (1), 1 (2)	arom. H, 7a (1), 2-CH_3 (2)
8-CO_2H (1)	175.1	–	–	–
**1 (2)**	**176.0**	**–**	**–**	**–**
2 (2)	43.9	2.34 (dq, $J_{2,2\text{-}CH3}$ = 6.8, $J_{2,3}$ = 9.7 Hz)	1 (2), 3 (2), 2-CH_3 (2)	arom. H, 8 (1), 2-CH_3 (2), 3 (2), 5a (2)/6b (2)
2-CH_3 (2)	14.3	1.24 (d)	1 (2), 2 (2), 3 (2)	8 (1), 2 (2), 3 (2), 4 (3), (3)/(4)-N-CH_3
3 (2)	85.6	3.49 (dd, $J_{3,4}$ = 4.6 Hz)	2 (2), 2-CH_3 (2), 3-OCH_3 (2), 4 (2), 5 (2)	n.d.
3-OCH_3 (2)	61.5	3.43 (s)	3 (2)	n.d.
4 (2)	59.5	3.21 (ddd, $J_{4,5a}$ = 6.5, $J_{4,5b}$ = 7.6 Hz)	n.d.	arom. H, 3 (2), 5b (2), 7a (2), 2 (3)
5 (2)	25.4	1.78 (H-5a, dddd, $J_{5a,6a}$ = 4.6, $J_{5a,6b}$ = 6.5, $J_{5a,5b}$ = –12.4 Hz)<br>1.44 (H-5b, dddd, $J_{5b,6a}$ = 6.3, $J_{5b,6b}$ = 8.0 Hz)	3 (2), 6 (2), 7 (2)	n.d.
6 (2)	23.0	1.93 (H-6a, dddd, $J_{6a,7a}$ = 7.8, $J_{6a,7b}$ = 8.2, $J_{6a,6b}$ = –12.8 Hz)<br>1.74 (H-6b, dddd, $J_{6b,7a}$ = 5.8, $J_{6b,7b}$ = 7.5 Hz)	5 (2), 7 (2)	n.d.
7 (2)	47.5	3.74 (H-7a, ddd, $J_{7a,7b}$ = –11.5 Hz)<br>3.31 (H-7b, ddd)	5 (2), 6 (2), 1 (3)	7a (2); 6b (2), 7b (2)<br>7b (2): 5a (2) , 6a (2), 7a (2)

**1 (3)**	**171.7**	–	–	–
2 (3)	35.6	2.55 (H-2a, d, $J_{2a,2b}$ = –15.7 Hz)<br>2.39 (H-2b, dd, $J_{2b,3}$ = 10.0 Hz)	1 (3), 3 (3)	2b (3): arom. H, 3 (2), 2a (3), 3 (3), 4 (3), (3)/(4)-N-CH_3
3 (3)	77.8	4.19 (ddd, $J_{3,5}$ = –1.6, $J_{3,4}$ = 5.4 Hz)	1 (3), 2 (3), 4 (3)	2 (3), aliphatic
3-OCH_3 (3)	57.9	3.41 (s)	3 (3)	n.d.
4 (3)	57.6	4.76 (dd, $J_{4,5}$ = 10.2 Hz)	3 (3), 5 (3), 1 (4)	2 (3), 5 (3), 6a (3), 7 (3), (3)/(4)-N-CH_3, aliphatic
5 (3)	~ 32.4	1.88 ddddq, $J_{5,6a}$ = 6.5, $J_{5,6b}$ = 6.5, $J_{5,5\text{-}CH3}$ = 6.5 Hz)	n.d.	2 (3), 3 (3), 4 (3), 6a (3), 7 (3), (3)/(4)-N-CH_3, aliphatic
5-CH_3 (3)	15.0	1.02 (d)	n.d.	n.d.
6 (3)	25.7	1.38 (H-6a ddq, $J_{6a,7}$ = 7.3, $J_{6a,6b}$ = –12.0 Hz)<br>~ 1.04 (H-6b, ddq, $J_{6b,7}$ = 7.5 Hz)	7 (3)	n.d.
7 (3)	9.9	0.89 (dd)	5 (3), 6 (3)	3 (3), 4 (3), 5 (3), 6a (3), (3)/(4)-N-CH_3, aliphatic
**(3)/(4)-N-CH_3**	**32.2**	**3.23 (s)**	4 (3), 1 (4)	arom. H, 2 (3), 3 (3), 4 (3), 5 (3), 6 (3), 7 (3), 2 (4), 3 (4), aliphatic
**1 (4)**	**173.9**	–	–	–
2 (4)	55.8	4.86 (d, $J_{2,3}$ = 8.5 Hz)	1 (4), 3 (4), 4 (4), 3-CH_3 (4), 1 (5)	(3)/(4)-N-CH_3, 3 (4), 3-CH_3 (4), 2 (5), (5)-N-CH_3, aliphatic
3 (4)	30.3	2.18 (dqq, $J_{3,3\text{-}CH3}$ = 6.8, $J_{3,4}$ = 6.9 Hz)	1 (4), 2 (4), 4 (4), 3-CH_3 (4)	arom. H, (3)/(4)-N-CH_3, 2 (4), aliphatic,
3-CH_3 (4)	18.4	1.13 (d)	2 (4), 3 (4), 4 (4)	arom H., (3)/(4)-N-CH_3, 2 (4), 3 (4), (5)-N-CH_3, aliphatic
4 (4)	~ 17.9	1.07 (d)	n.d.	n.d.
**1 (5)**	**167.2**	–	–	–
2 (5)	67.0	3.84 (d, $J_{2,3}$ = 5.8 Hz)	1 (5), 3 (5), 4 (5), 3-CH_3 (5), (5)-N-CH_3	2 (4), 3 (4), 3 (5), (5)-N-CH_3, aliphatic
3 (5)	~ 29.9	2.27(dqq, $J_{3,3\text{-}CH3}$ = 6.9, $J_{3,4}$ = 6.9 Hz)	1 (5), 2 (5), 4 (5), 3-CH_3 (5)	n.d.
3-CH_3 (5)	18.0 – 17.0	1.10 (d)	n.d.	n.d.
4 (5)	18.0 – 17.0	~ 1.03 (d)	n.d.	n.d.
**(5)-N-CH_3**	**~ 32.2**	**2.78 (s)**	2 (5)	2 (4), 2 (5), 3 (5), aliphatic

[A] The HMBC and ROE correlations were not determined in crowded areas of the spectrum where the uncertainty was high, if the ROE-correlations were in the aliphatic region it is mentioned in the table.

Supporting Table 4. Summary of the NMR results of compound **2B** (*trans*-conformer of Cl-MMAF) measured at 37°C in D_2O with a Bruker 850 MHz instrument. Chemical shifts are expressed in ppm using solvent residual peaks as an internal reference. Coupling patterns are given as d (doublet), t (triplet), q (quartet), m (multiplet) etc. and the approximate coupling constants are provided only once when first encountered.

POSITION	δ ^{13}C	δ ^{1}H (*J*, HZ)	HMBC (H → C)-CORRELATIONS[A]	ROESY CORRELATIONS[A]
**1′ (1′)**	**136.0**	–	–	–
2′ (1′)	130.6	7.35 – 7.30 (m)	arom. C	7′ (1′), 8′ (1′), 4′ (2′), 5′ (2′)
3′ (1′)	128.8	7.44 – 7.35 (m)	arom. C	2′-CH_3 (2′), 3′ (2′), 5′ (2′), 7′a (2′), 2′ (3′), 3′ (3′), aliphatic
4′ (1′)	131.9	–	–	–
5′ (1′)	128.8	7.44 – 7.35 (m)	arom. C	2′-CH_3 (2′), 3′ (2′), 5′ (2′), 7′a (2′), 2′ (3′), 3′ (3′), aliphatic
6′ (1′)	130.6	7.35 – 7.30 (m)	arom. C	7′ (1′), 8′ (1′), 4′ (2′), 5′ (2′)
7′ (1′)	36.0	3.49 (H-7′a, dd, $J_{7'a,8'}$ = 4.7, $J_{7'a,7'b}$ = –14.0 Hz)<br>2.91 (H-7′b, dd, $J_{7'b,8'}$ = 11.7 Hz)	1′ (1′), 8′-CO_2H (1′), 8′ (1′)	7′a ↔ 7′b<br>7′b (1′): arom. H, 8′ (1′)
8′ (1′)	52.8	5.00 (dd)	1′ (1′), 7′ (1′), 8′-CO_2H (1′), 1′ (2′)	2′ (2′), 2′-CH_3 (2′), 3′ (2′), 4′ (2′)
8′-CO_2H (1)	175.1	–	–	–
**1′ (2′)**	**176.2**	–	–	–
2′ (2′)	44.2	2.28 (dq, $J_{2'a,2'\text{-}CH3}$ = 6.8, $J_{2',3'}$ = 9.7 Hz)	1′ (2′), 3′ (2′), 2′-CH_3 (2′)	n.d.
2′-CH_3 (2′)	13.8	1.18 (d)	1′ (2′), 2′ (2′), 3′ (2′)	arom. H, 8′ (1′), 2′ (2′), 3′ (2′), 4′ (2′)
3′ (2′)	82.5	3.76 (dd, $J_{3',4'}$ = 1.8 Hz)	1′ (2′), 2′ (2′), 2′-CH_3 (2′), 3′-OCH_3 (2′), 4′ (2′), 5′ (2′)	arom. H, 8′ (1′), 2′ (2′), 2′-CH_3 (2′), 5′a (2′), 6′a (2′), 2′b (3′)
3′-OCH_3 (2′)	61.1	3.39 (s)	3′ (2′)	2′ (2′), 2′-CH_3 (2′), 3′ (2′), 4′ (2′), 5′a (2′), 6′a (2′), 2′b (3′), 3′ (3′)
4′ (2′)	59.0	2.82 (ddd, $J_{4',5'b}$ = 5.9, $J_{4',5'a}$ = 6.3 Hz)	3′ (2′), 5′ (2′), 7′ (2′), 1′ (3′)	arom. H, 8′ (1′), 2′ (2′),2′-CH_3 (2′), 2′ (2′), 3′ (2′), 5′ (2′), 7′a (2′), 3′ (3′)
5′ (2′)	24.2	1.66 (H-5′a, dddd, $J_{5'a,6'b}$ = 5.6, $J_{5'a,6'a}$ = 7.7, $J_{5'a,5'b}$ = –12.7 Hz)<br>1.45 (H-5′b, dddd, $J_{5'b,6'b}$ = 6.8, $J_{5'b,6'a}$ = 7.0 Hz)	3′ (2′), 4′ (2′), 7′ (2′)	5′a ↔ 5′b<br>5′a (2′): 2′ (2′), 3′ (2′), 4′ (2′), 5′b (2′), 6′a (2′)

6′ (2′)	24.2	1.92 (H-6′a, dddd, $J_{6'a,7'b}$ = 5.6, $J_{6'a,7'a}$ = 6.3, $J_{6'a,6'b}$ = –13.7 Hz)<br>1.76 (H-6′b, dddd, $J_{6'b,7'b}$ = 6.8, $J_{6'b,7'a}$ = 7.0 Hz)	n.d.	n.d.
7′ (2′)	48.3	3.60 (H-7′a, ddd, $J_{7'a,7'b}$ = –10.5 Hz)<br>3.43 (H-7′b, ddd)	4′ (2′), 5′ (2′), 6′ (2′),	7′a (2′): 4′ (2′), 5′b (2′), 6′ (2′), 2′ (3′), (3′)/(4′)-N-CH_3, aliphatic
**1′ (3′)**	**170.6**	**–**	**–**	–
2′ (3′)	36.8	2.62 (H-2′a, d, $J_{2'a,3'}$ = 1.7, $J_{2'a,2'b}$ = –16.0 Hz)<br>2.55 (H-2′b, dd, $J_{2'b,3'}$ = 9.3 Hz)	1′ (3′), 3′ (3′)	2′a (3′): 7′a (2′), 2′b (3′), 3′ (3′), 5′ (3′), (3′)/(4′)-N-CH_3, aliphatic<br>2′b (3′): 2′a (3′), 3′ (3′), 5′ (3′), (3′)/(4′)-N-CH_3, aliphatic
3′ (3′)	77.9	4.17 (ddd, $J_{3',4'}$ = 5.0 Hz)	1′ (3′), 4′ (3′)	3′ (2′), 2′a (3′), 5′ (3′), 6′a (3′), (3′)/(4′)-N-CH_3, aliphatic
3′-OCH_3 (3′)	57.6	3.36 (s)	3′ (3′)	2′b (3′), 3′ (3′), 4′ (3′), 5′ (3′), 6′a (3′), aliphatic
4′ (3′)	57.9	4.71 (dd, $J_{4',5'}$ = 4.7 Hz)	n.d.	2′ (3′), 5′ (3′), 6′a (3′), 7′ (3′), (3′)/(4′)-N-CH_3, aliphatic
5′ (3′)	~ 32.4	1.88 (dddq, $J_{5',5'\text{-}CH3}$ = 6.5, $J_{5',6'a}$ = 6.5, $J_{5',6'b}$ = 6.5 Hz)	n.d.	2′ (3′), 3′ (3′), 4′ (3′), 6′a (3′), 7′ (3′), (3′)/(4′)-N-CH_3, aliphatic
5′-CH_3 (3′)	15.5	1.08 (d)	n.d.	n.d.
6′ (3′)	25.7	1.41 (H-6′a, ddq, $J_{6'a,7'}$ = 7.5, $J_{6',6'b}$ = –12.0 Hz)<br>~ 1.04 (H-6′b, ddq, $J_{6'b,7'}$ = 7.4 Hz)	7′ (3′)	n.d.
7′ (3′)	10.1	0.90 (dd)	5′ (3′), 6′ (3′)	3′ (3′), 4′ (3′), 5′ (3′), 6′a (3′), (3′)/(4′)-N-CH_3, 3′ (5′), aliphatic
**(3′)/(4′)-N-CH_3**	**32.4**	**3.18 (s)**	4′ (3′), 1′ (4′)	2′ (3′), 3′ (3′), 4′ (3′), 5′ (3′), 6′ (3′), 7′ (3′), 2′ (4′), aliphatic
**1′ (4′)**	**174.2**	**–**	**–**	–
2′ (4′)	55.8	4.81 (d, $J_{2',3'}$ = 9.0 Hz)	1′ (4′), 3′ (4′), 3′-CH_3 (4′), 4′ (4′), 1′ (5′)	(3′)/(4′)-N-CH_3, 3′ (4′), aliphatic, (5′)-N-CH_3, 2′ (5′)
3′ (4′)	30.2	2.14 (dqq, $J_{3',4'}$ = 6.5, $J_{3',3'\text{-}CH3}$ = 7.0 Hz)	2′ (4′), 3′-CH_3 (4′), 4′ (4′)	(3′)/(4′)-N-CH_3, 2′ (4′), aliphatic
3′-CH_3 (4′)	18.2	1.07 (d)	n.d.	n.d.
4′ (4′)	18.0 – 17.0	1.05 (d)	n.d.	n.d.

**1′ (5′)**	**167.2**	**–**	**–**	–
2′ (5′)	67.0	3.84 (d, $J_{2′,3′}$ = 5.5 Hz)	1′ (5′), 3′ (5′), 3′-CH_3 (5′), 4′ (5′), (5′)-N-CH_3	2′ (4′), 3′ (4′), 3′ (5′), aliphatic, (5′)-N-CH_3
3′ (5′)	~ 29.9	2.27 (dqq, $J_{3′,4′}$ = 6.5, $J_{3′,3′\text{-}CH3}$ = 6.8 Hz)	1′ (5′), 2′ (5′), 3′-CH_3 (5′), 4′ (5′)	n.d.
3′-CH_3 (5′)	18.0 – 17.0	1.10 (d)	2′ (5′), 3′ (5′), 4′ (5′)	n.d.
4′ (5′)	18.0 – 17.0	~ 1.03 (d)	n.d.	n.d.
**(5′)-N-CH_3**	**~ 32.3**	**2.76 (s)**	2′ (5′)	2′ (4′), 2′ (5′), 3′ (5′), aliphatic

[A] The HMBC and ROE correlations were not determined in crowded areas of the spectrum where the uncertainty was high, if the ROE-correlations were in the aliphatic region it is mentioned in the table.

2. Selected NMR spectra

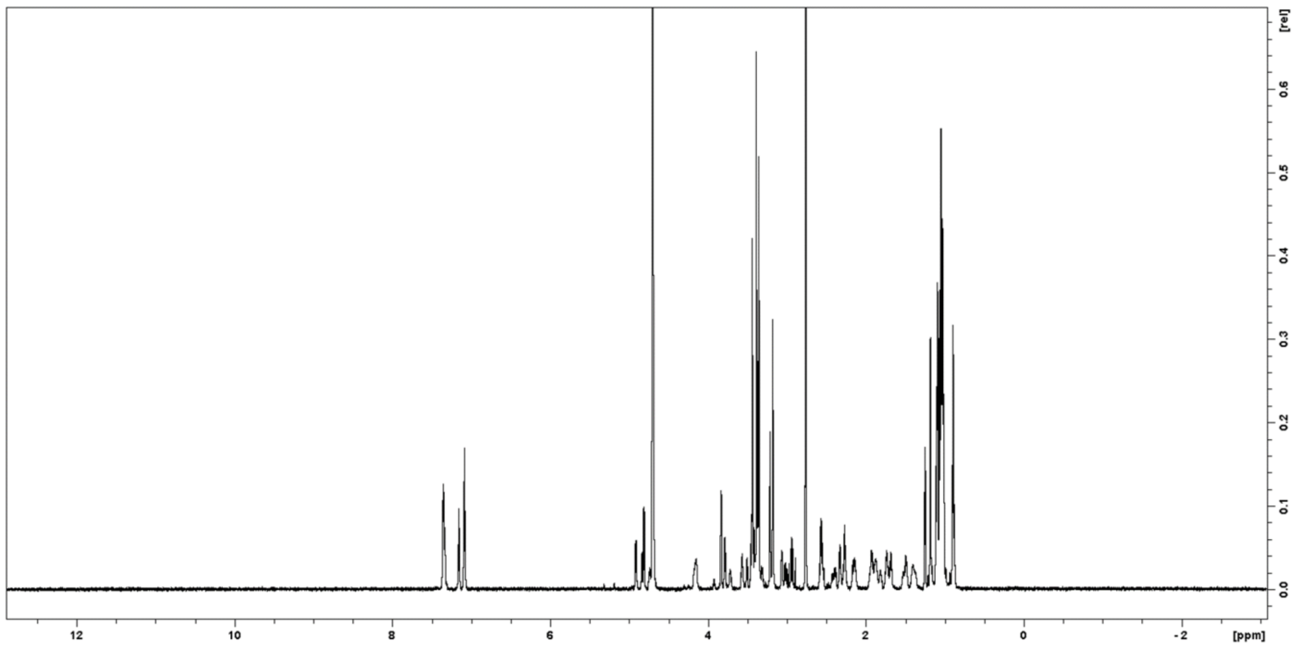

Supporting Figure 1. ^1H NMR spectrum of **1** recorded at 37 °C in D_2O (850 MHz).

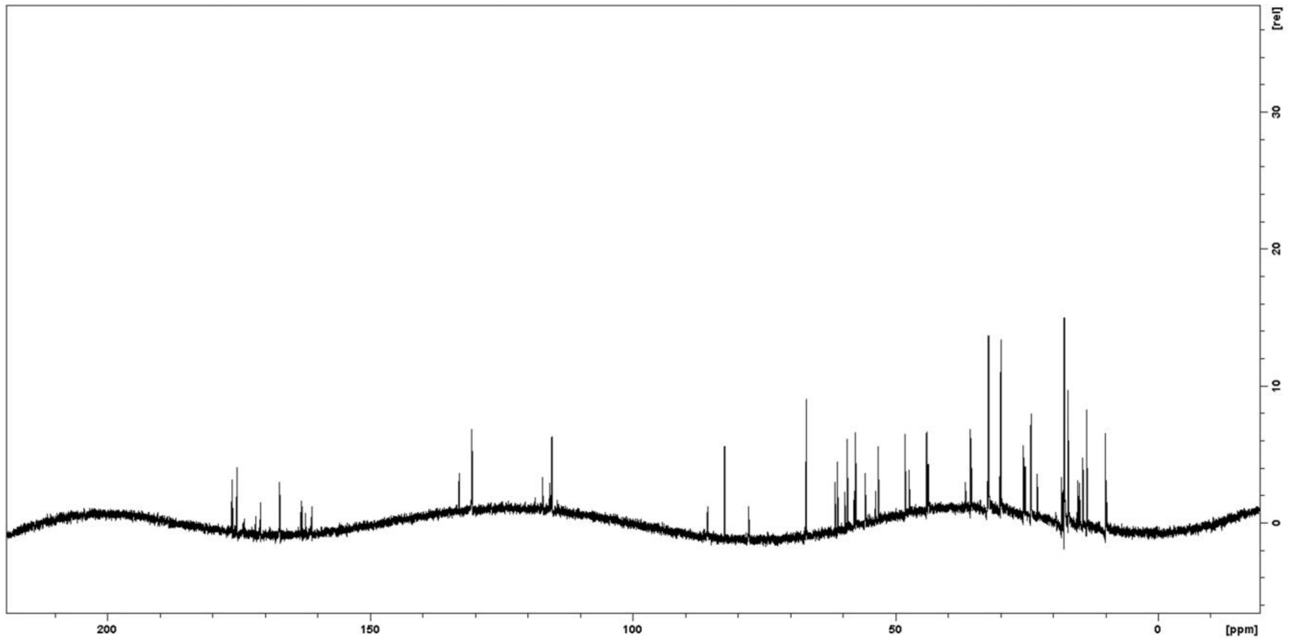

Supporting Figure 2. ^{13}C NMR spectrum of **1** recorded at 37 °C in D_2O (213 MHz).

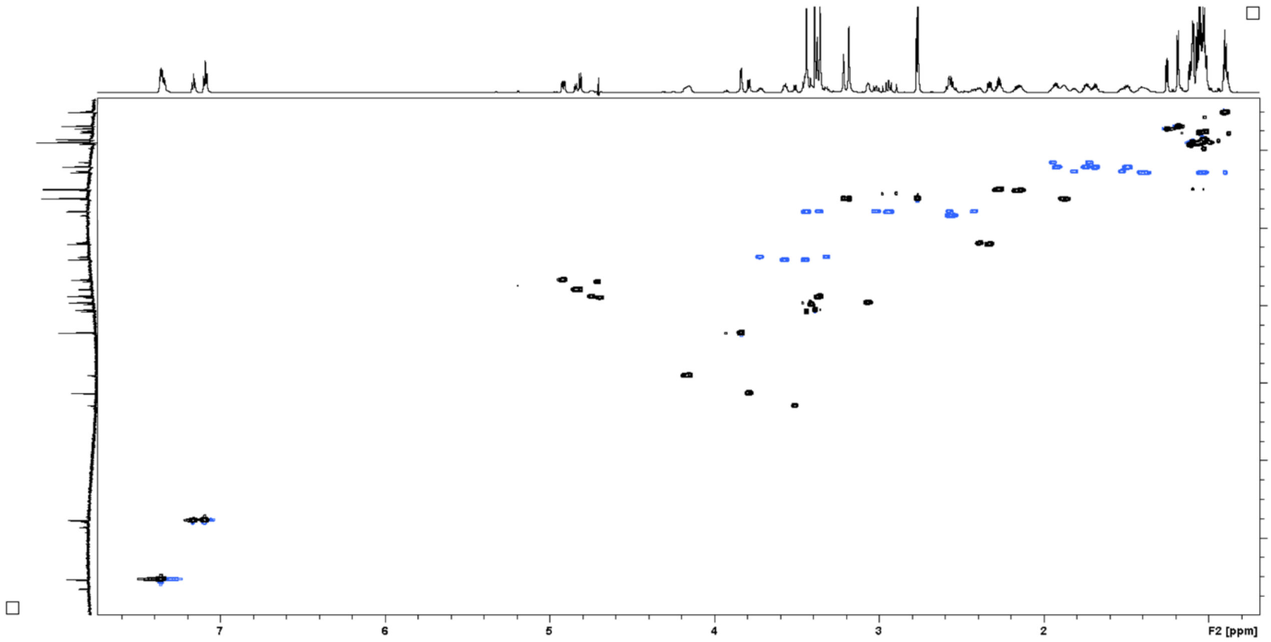

Supporting Figure 3. EdHSQC spectrum of **1** recorded at 37 °C in D_2O (850 MHz).

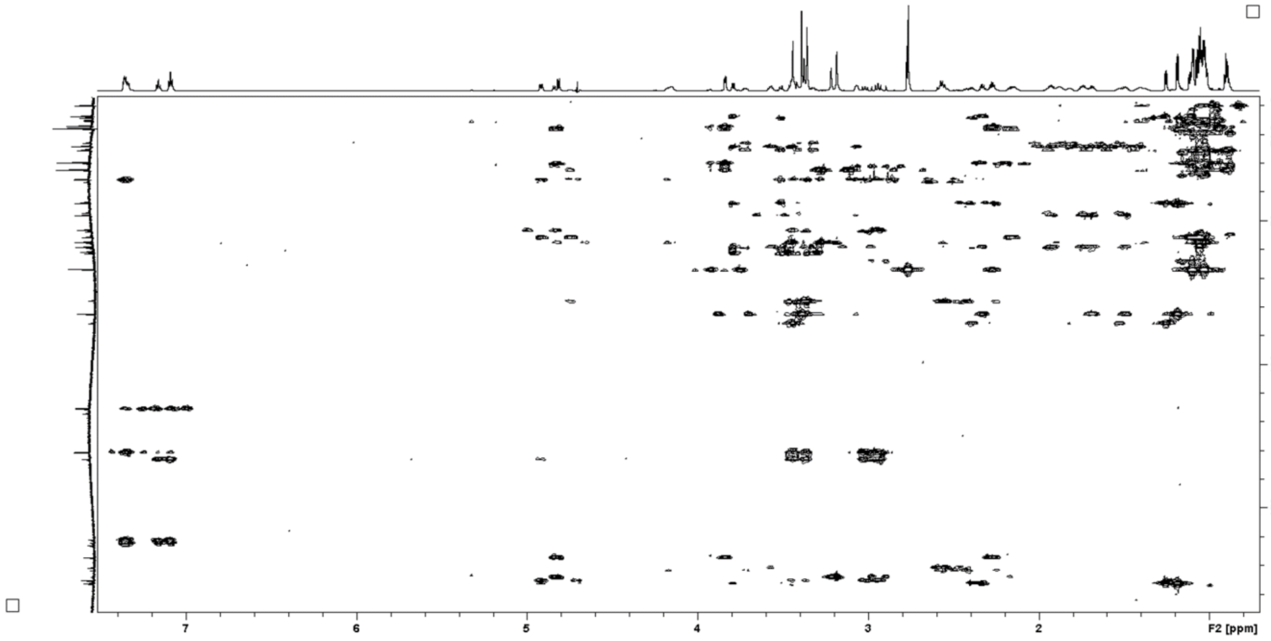

Supporting Figure 4. 2D TOCSY spectrum of **1** recorded at 37 °C in D_2O (850 MHz).

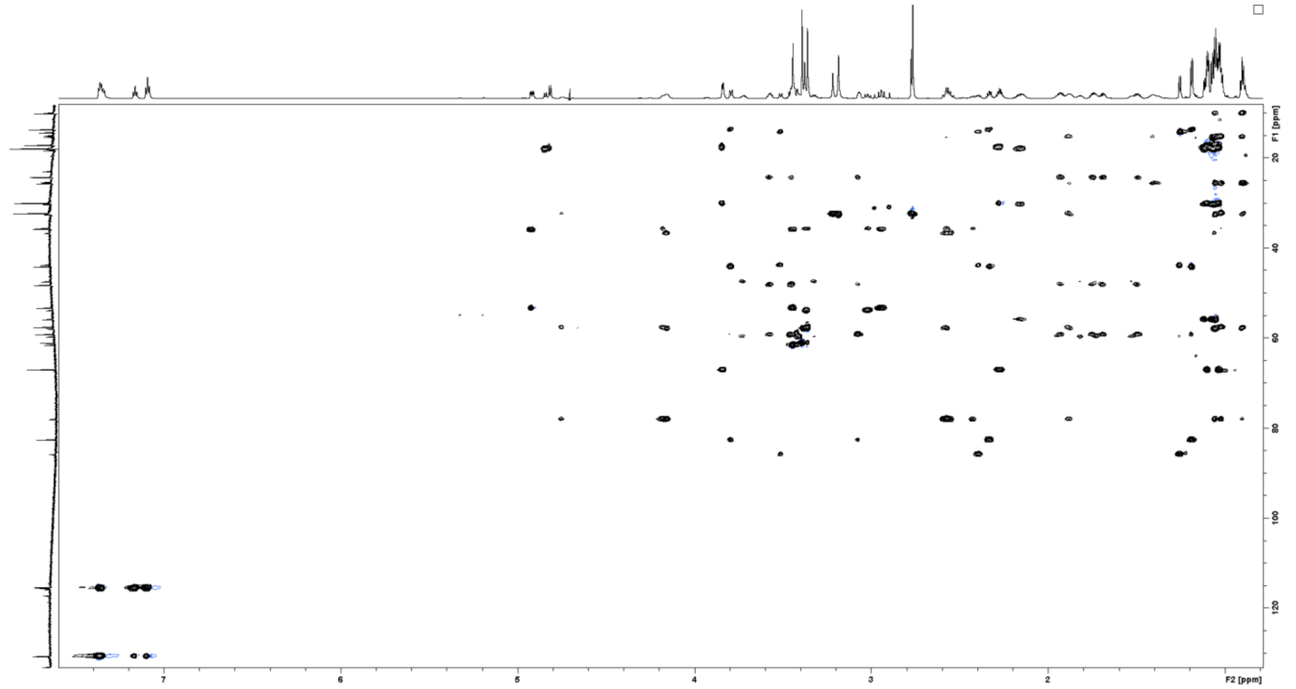

Supporting Figure 5. HSQC-TOCSY spectrum of **1** recorded at 37 °C in D_2O (850 MHz).

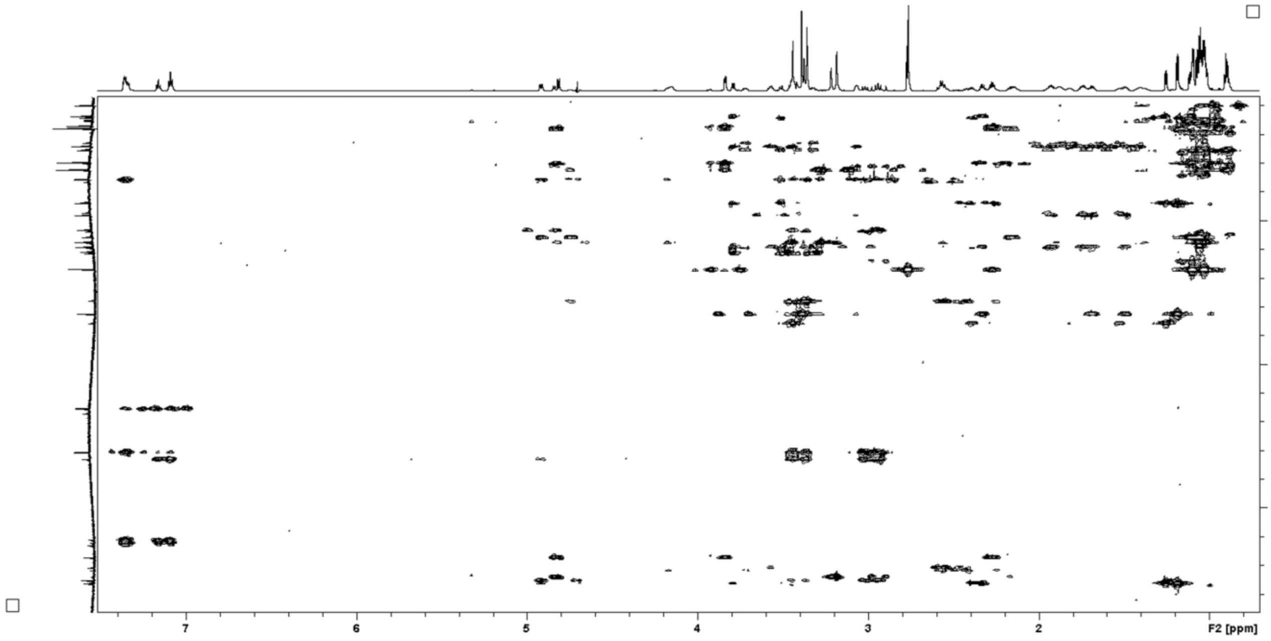

Supporting Figure 6. HMBC spectrum of **1** recorded at 37 °C in D_2O (850 MHz).

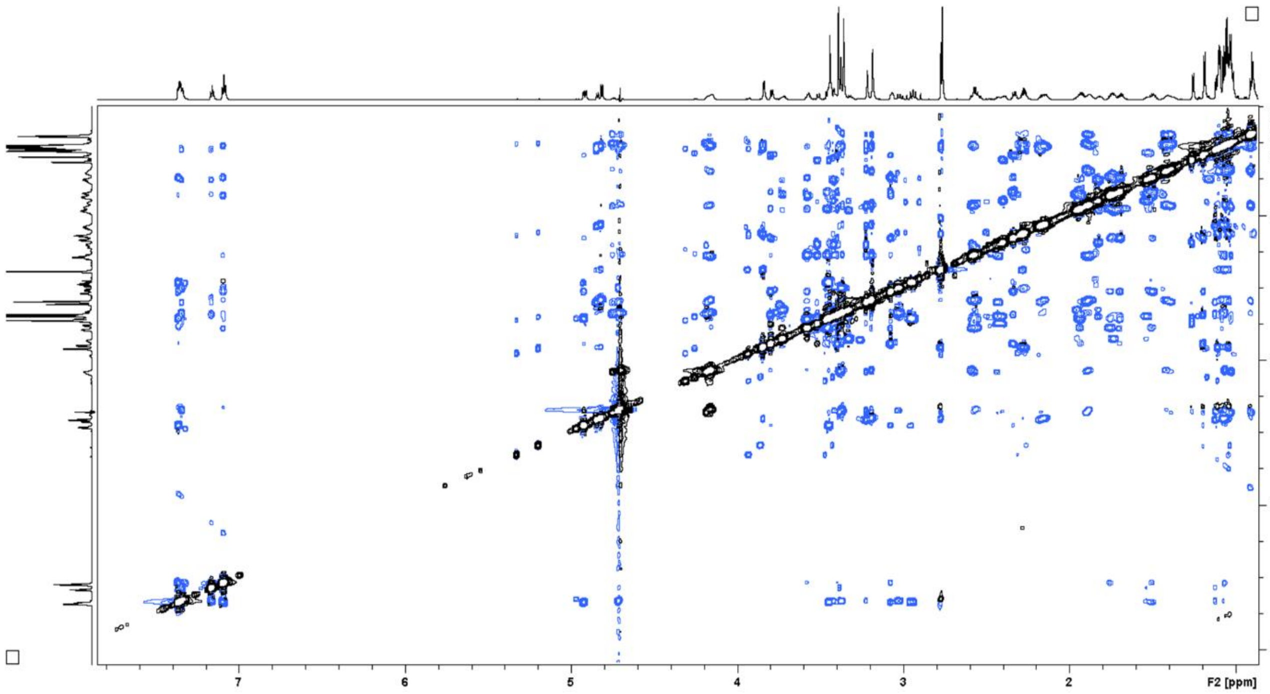

Supporting Figure 7. ROESY spectrum of **1** recorded at 37 °C in D_2O (850 MHz).

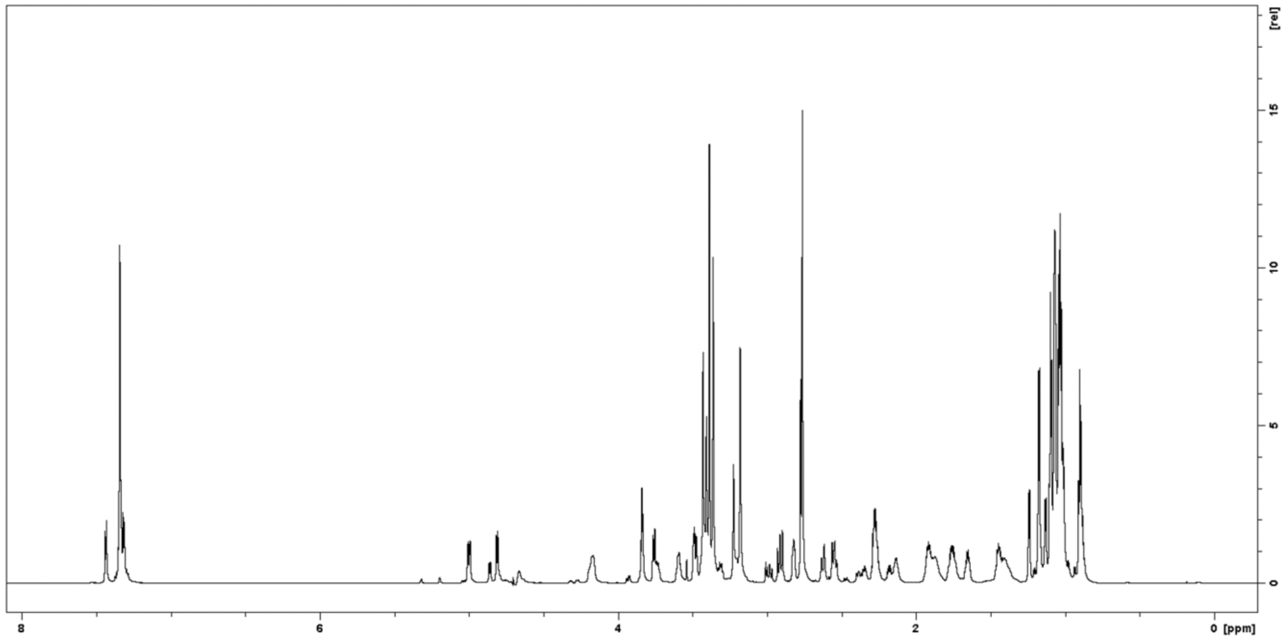

Supporting Figure 8. ^{1}H NMR spectrum of **2** recorded at 37 °C in D_2O (850 MHz).

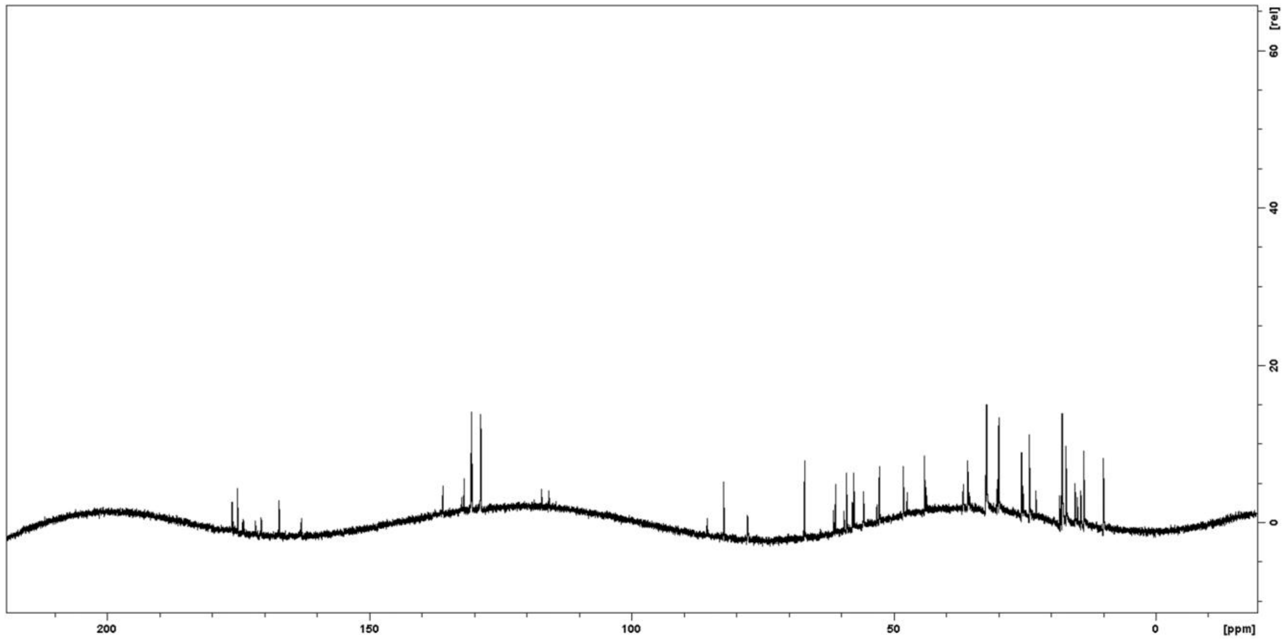

Supporting Figure 9. ^{13}C NMR spectrum of **2** recorded at 37 °C in D_2O (213 MHz).

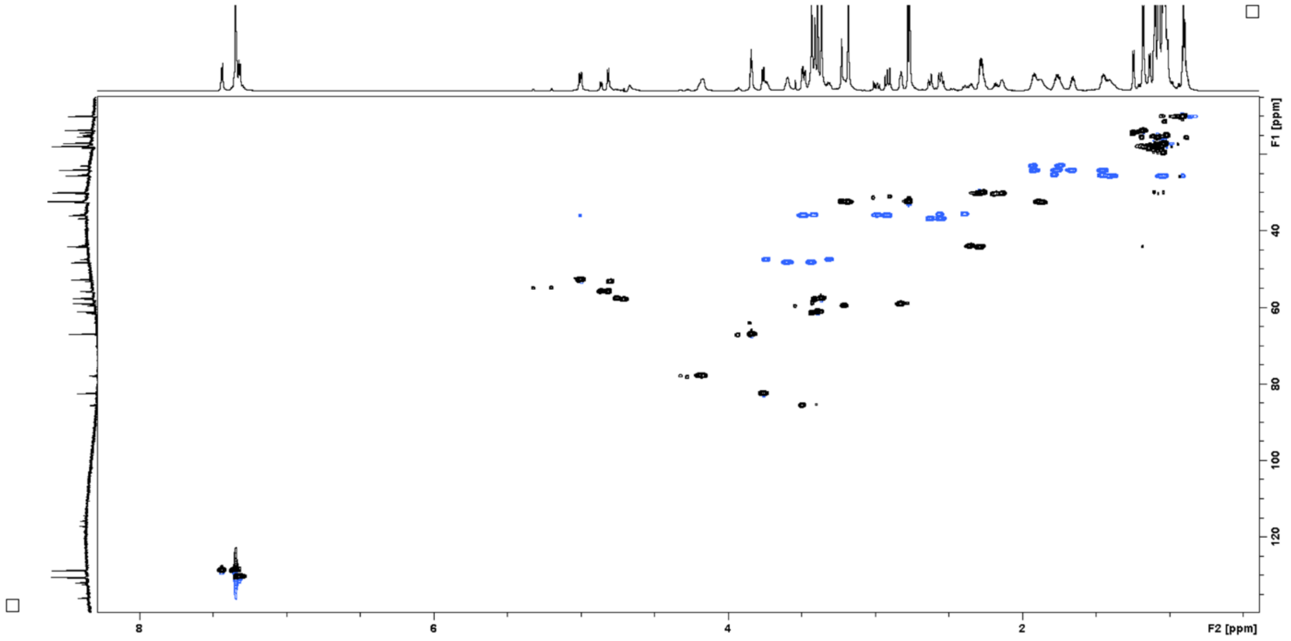

Supporting Figure 10. EdHSQC spectrum of **2** recorded at 37 °C in D_2O (850 MHz).

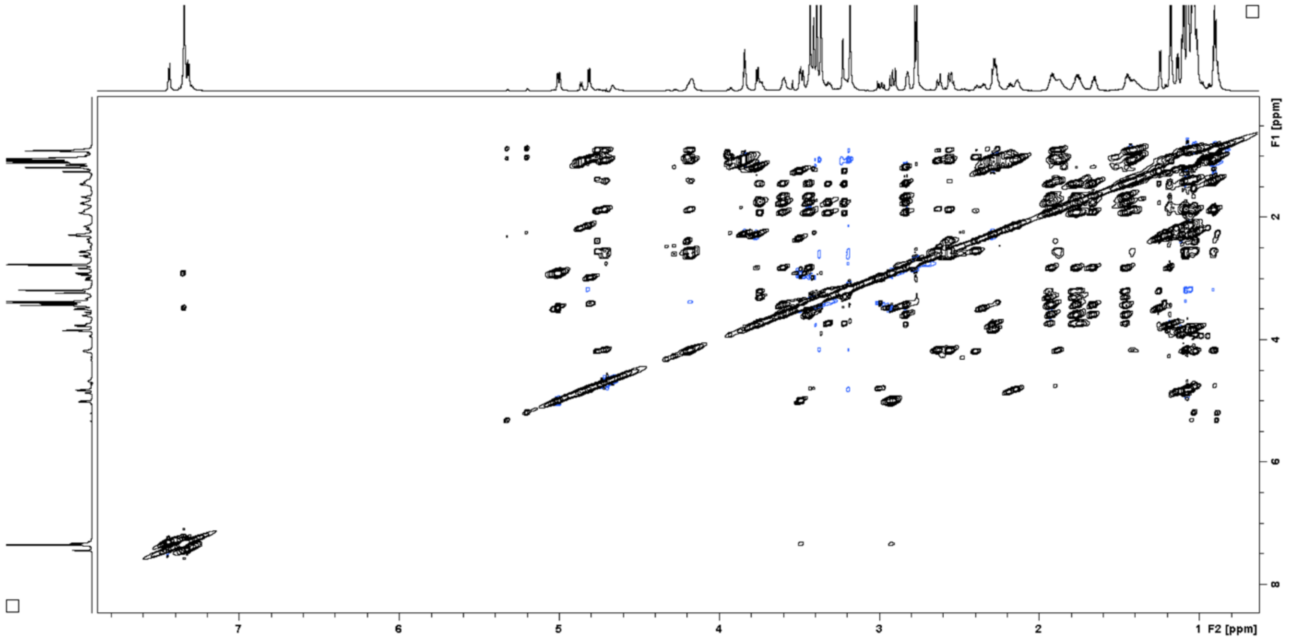

Supporting Figure 11. 2D TOCSY spectrum of **2** recorded at 37 °C in D_2O (850 MHz).

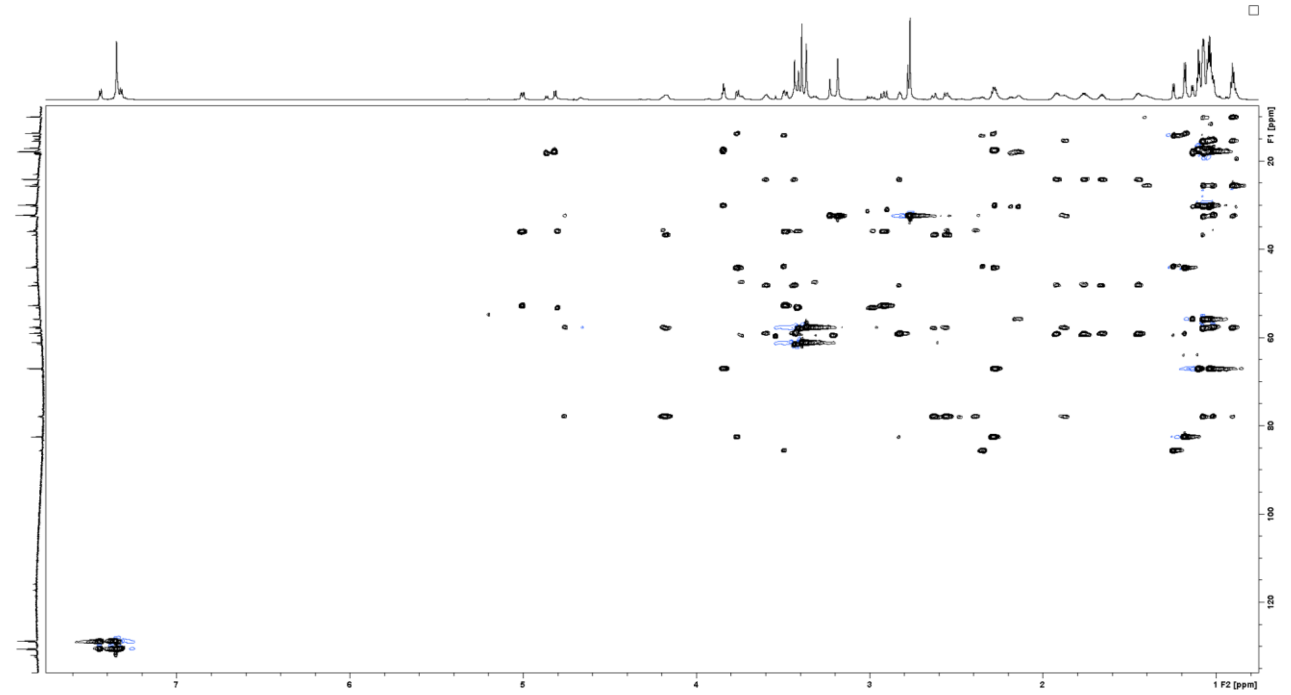

Supporting Figure 12. HSQC-TOCSY spectrum of **2** recorded at 37 °C in D_2O (850 MHz).

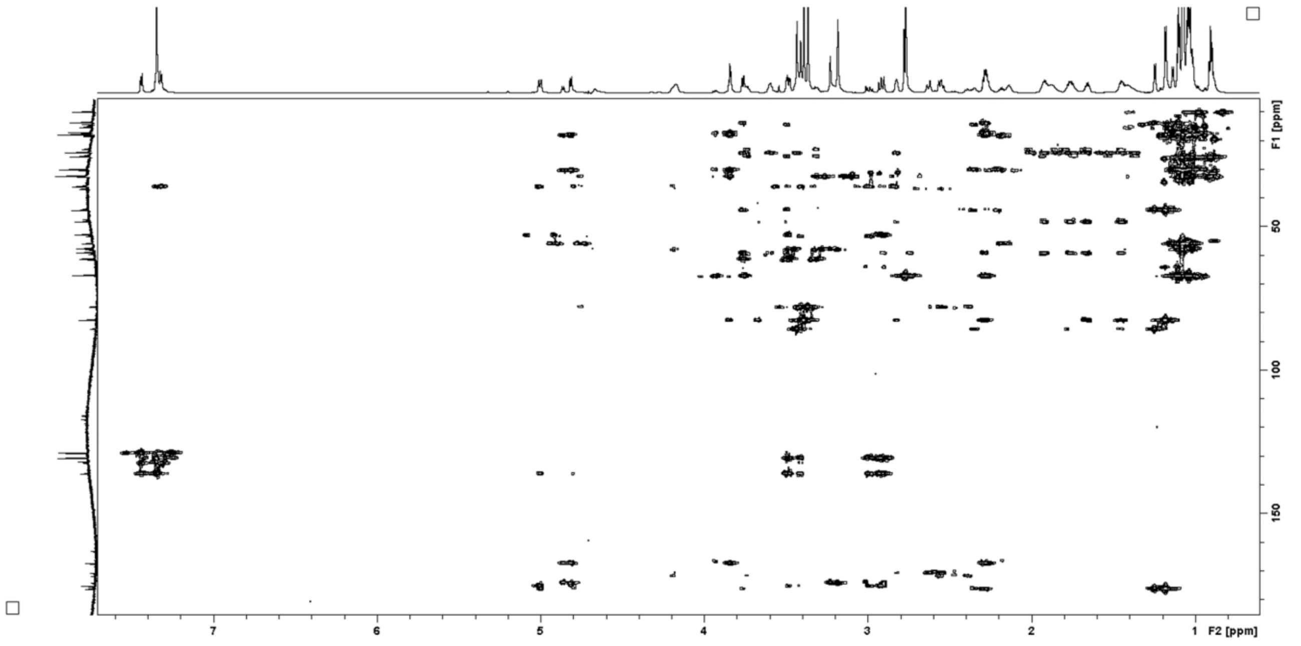

Supporting Figure 13. HMBC spectrum of **2** recorded at 37 °C in D_2O (850 MHz).

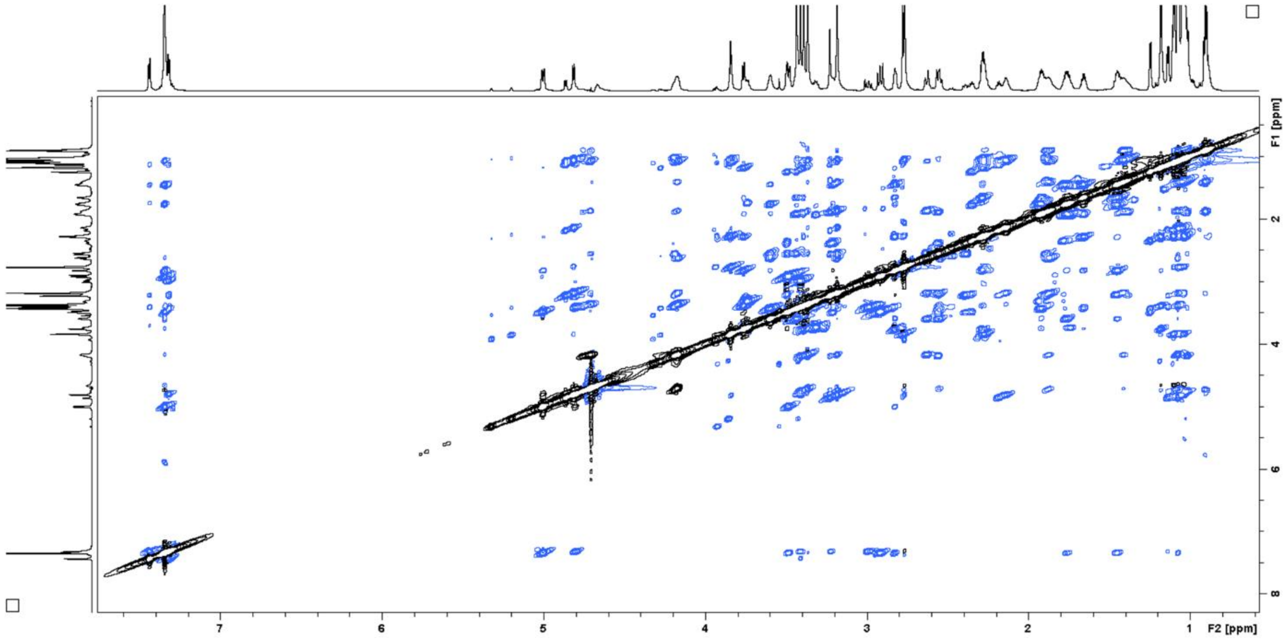

Supporting Figure 14. ROESY spectrum of **2** recorded at 37 °C in D_2O (850 MHz).

3. Supporting material related to cytotoxicity studies

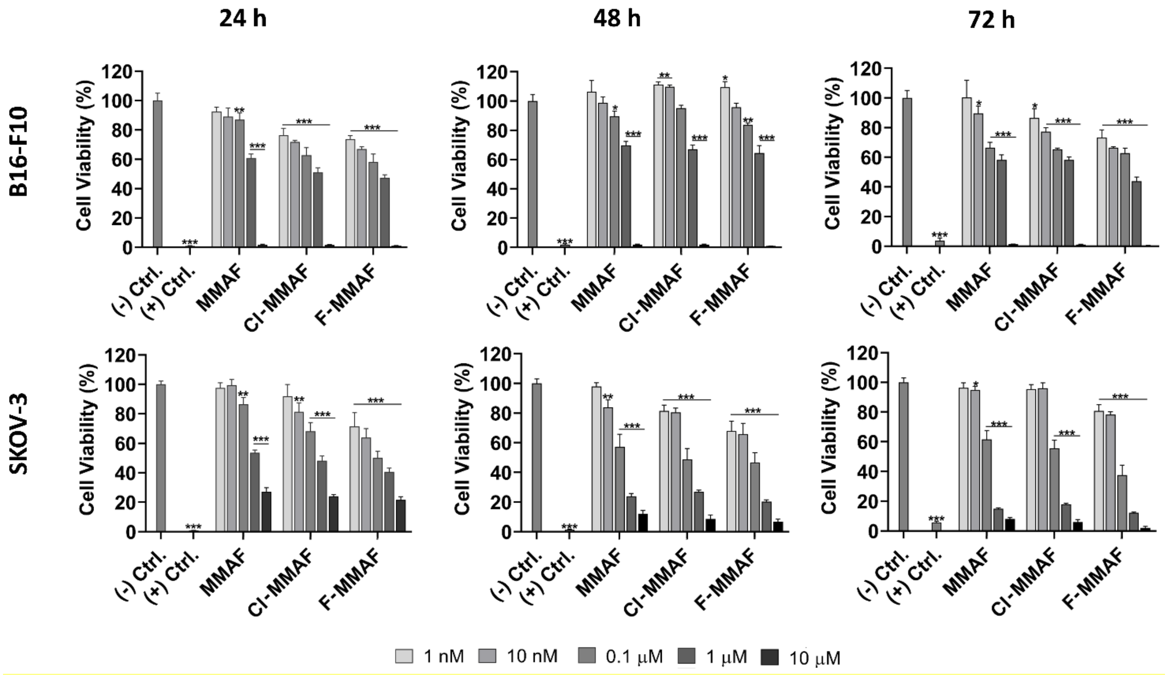

Supporting Figure 15. Cell cytotoxicity studies in murine B16-F10 and human SKOV3 cancer cells after incubation with negative (cell culture medium) and positive (1% Triton X-100) controls for cytotoxicity, and the auristatins; MMAF, **1** (F-MMAF), **2** (Cl-MMAF) at concentrations of 1 nM, 10 nM, 0.1 µM, 1 µM, and 10 µM for 24, 48, and 72 h. Columns represent the mean ± s.d. (n=4). The statistical significance of the difference in comparison to the negative control was determined using unpaired Student's *t*-test with the significance set at *p<0.05, **p<0.01, and ***p<0.001.

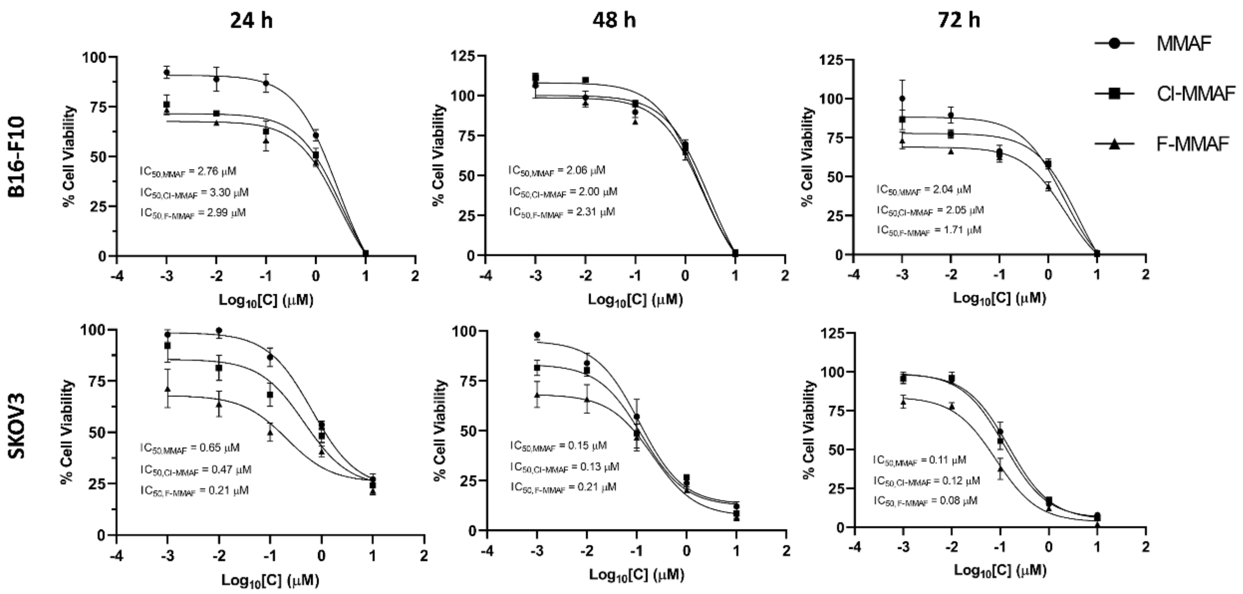

Supporting Figure 16. Dose–response diagrams with IC_{50} values of MMAF, **1** (F-MMAF), and **2** (Cl-MMAF) in murine B16-F10 and human SKOV3 cancer cell lines at 24, 48, and 72 h. The curves are the representation of nonlinear regression fitting where the concentrations are plotted in logarithmic scale against percent of viable cells. Data is expressed as mean ± s.d. (n=4).
